# CAR-T cell therapy for cancer: current challenges and future directions

**DOI:** 10.1038/s41392-025-02269-w

**Published:** 2025-07-04

**Authors:** Inés Zugasti, Lady Espinosa-Aroca, Klaudyna Fidyt, Vladimir Mulens-Arias, Marina Diaz-Beya, Manel Juan, Álvaro Urbano-Ispizua, Jordi Esteve, Talia Velasco-Hernandez, Pablo Menéndez

**Affiliations:** 1 Hematology Department, Hospital Clínic de Barcelona, Institut d’Investigacions Biomèdiques August Pi i Sunyer (IDIBAPS), Barcelona, Spain; 2 Josep Carreras Leukemia Research Institute, Barcelona, Spain; 3 Red Española de Terapias Avanzadas (TERAV), Instituto de Salud Carlos III, Madrid, Spain; 4 Department of Immunology, Centre de Diagnòstic Biomèdic (CDB), Hospital Clínic de Barcelona, Barcelona, Spain; 5 Departments of Biomedical Sciences and Medicine and Health Sciences, University of Barcelona, Barcelona, Spain; 6 Fundació de Recerca Clínic Barcelona-Institut d’Investigacions Biomèdiques August Pi i Sunyer, Barcelona, Spain; 7 Immunotherapy Joint Platform of Hospital Sant Joan de Deu and Hospital Clínic de Barcelona, Barcelona, Spain; 8 Centro de Investigación Biomédica en Red de Cáncer (CIBERONC), Madrid, Spain; 9 Institució Catalana de Recerca i Estudis Avançats (ICREA), Barcelona, Spain; 10 Pediatric Cancer Centre Barcelona-Institut de Recerca Sant Joan de Deu (PCCB-SJD), Barcelona, Spain

**Keywords:** Haematological cancer, Immunotherapy

## Abstract

Chimeric antigen receptor T (CAR-T) cell therapies have transformed the treatment of relapsed/refractory (R/R) B-cell malignancies and multiple myeloma by redirecting activated T cells to CD19- or BCMA-expressing tumor cells. However, this approach has yet to be approved for acute myeloid leukemia (AML), the most common acute leukemia in adults and the elderly. Simultaneously, CAR-T cell therapies continue to face significant challenges in the treatment of solid tumors. The primary challenge in developing CAR-T cell therapies for AML is the absence of an ideal target antigen that is both effective and safe, as AML cells share most surface antigens with healthy hematopoietic stem and progenitor cells (HSPCs). Simultaneously targeting antigen expression on both AML cells and HSPCs may result in life-threatening on-target/off-tumor toxicities such as prolonged myeloablation. In addition, the immunosuppressive nature of the AML tumor microenvironment has a detrimental effect on the immune response. This review begins with a comprehensive overview of CAR-T cell therapy for cancer, covering the structure of CAR-T cells and the history of their clinical application. It then explores the current landscape of CAR-T cell therapy in both hematologic malignancies and solid tumors. Finally, the review delves into the specific challenges of applying CAR-T cell therapy to AML, highlights ongoing global clinical trials, and outlines potential future directions for developing effective CAR-T cell-based treatments for relapsed/refractory AML.

## Introduction

Acute myeloid leukemia (AML) is the predominant form of leukemia in adults, with a median age at diagnosis of 68 years.^1,2^ It is primarily characterized by a high degree of complex clonal heterogeneity.^3,4^ For patients eligible for high-dose chemotherapy, treatment typically involves a combination of cytarabine and daunorubicin or idarubicin. Additionally, for those classified as intermediate or high risk according to the European Leukemia Net 2022 (ELN22)^5^ risk stratification, allogeneic hematopoietic stem cell transplantation (alloHSCT) is often performed following initial chemotherapy. However, elderly patients or those with comorbidities who are ineligible for alloHSCT are typically treated with low-intensity regimens.^5^ These regimens include venetoclax combined with hypomethylating agents (HMAs),^6,7^ low-dose cytarabine,^8,9^ or targeted molecules such as FLT3-directed tyrosine kinase inhibitors,^10,11^ as well as HMAs with IDH1/2 inhibitors.^12,13^

Despite the great efforts that have been made in recent years and the approval of new targeted therapies, relapsed/refractory (R/R) AML remains the leading cause of treatment failure. This challenging scenario occurs in 40-50% of patients younger than 60 years of age^2,14^ and in up to 80% of patients older than 65 years, many of whom are ineligible for alloHSCT or intensive chemotherapy.^15–17^ There is currently no standardized treatment protocol for these patients, and the 5-year overall survival (OS) rate is below 20%.^18^ Therefore, R/R AML remains an unmet clinical need, and further investigation is urgent. For patients who meet eligibility criteria, alloHSCT is the only potentially curative option in this setting.^2^

The success of alloHSCT and other cell therapy-based strategies, such as donor lymphocyte infusion (DLI), relies on the capacity of T and natural killer (NK) cells to target and eliminate leukemic cells, underscoring the sensitivity of AML to immunotherapy.^19^ This has prompted interest in novel immunotherapeutic approaches, which are currently being explored.^20^ For example, some clinical trials have tested immune checkpoint blockade strategies, including the anti-TIM3 monoclonal antibody (mAb) sabatolimab (NCT04266301), with limited results,^21^ or antibodies targeting CD47 (e.g., magrolimab, lemzoparlimab).^22^ A clinical trial is currently underway (NCT03113643) testing a recombinant protein consisting of interleukin (IL)-3 fused to a truncated diphtheria toxin payload that targets CD123 (tagraxofusp).^23^ Additional ongoing strategies include the use of menin inhibitors targeting the HOX/MEIS1 transcriptional program, in combination with chemotherapy, for KMT2A rearranged- or NPM1-mutated AML, with encouraging results.^24,25^

However, despite the excellent clinical outcomes observed with several chimeric antigen receptor T (CAR-T) cell products targeting CD19 or BCMA in the treatment of R/R B-cell malignancies and R/R multiple myeloma (MM), no CAR-T cell product has yet received regulatory approval for AML. This review offers an overview of the current development of CAR-T cell therapies for both hematologic and solid tumors, while examining the challenges associated with their application in AML, ongoing clinical trials, and future directions for optimizing CAR-T cell therapy in the treatment of AML.

## CAR-T cell structure

**Fig. 1:**
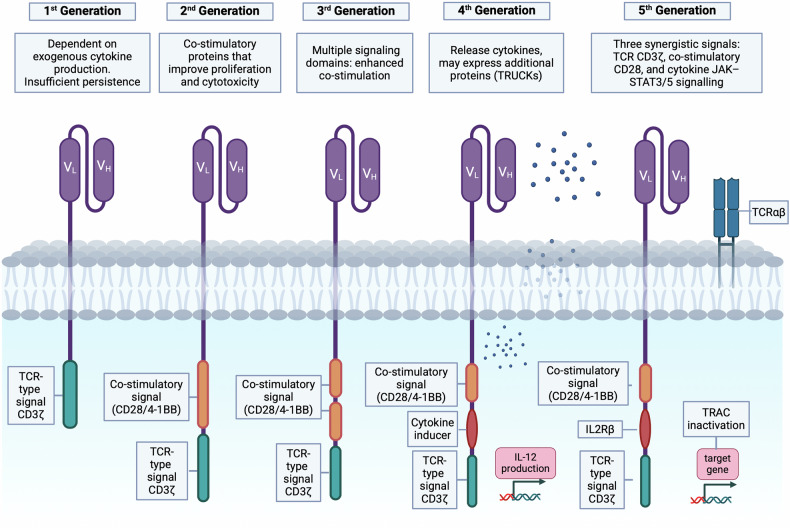
Structure of CARs. First-generation CARs consist of a ligand or scFv ectodomain and a CD3ζ TCR-type intracellular signal. Second-generation CARs contain a scFv extracellular domain and a co-stimulatory domain, 4-1BB or CD28. Third-generation CARs contain two co-stimulatory domains (usually 4-1BB and CD28). Fourth-generation CARs (TRUCKs) contain a domain encoding a specific cytokine or signal blocker/inducer. Fifth-generation CARs contain three synergistic co-stimulatory signals. This figure was created using Biorender.com

CARs are engineered receptors consisting of a combination of an endodomain, an anchoring transmembrane domain, and an ectodomain.^26,27^ The latter is a ligand-specific extracellular domain consisting of a single-chain variable-fragment (scFv) region and a hinge.^28^ The scFv is a fusion protein of the variable regions of the light and heavy chains of immunoglobulins linked by a short flexible peptide linker.^29^ The hinge, also known as a spacer, separates the binding units from the transmembrane domain.^30^ Most CAR-T cells are designed with immunoglobulin-like domain hinges, which provide flexibility in accessing the target antigen.^31,32^ The endodomain may consist of the intracellular T cell activation domain of CD3ζ as a single entity or by one or more intracellular co-stimulatory (or activation) domains.^33^ While the scFv provides antigen specificity, the co-stimulatory domains are key to the activation of effector T cells.^34^ CAR-T cells are classified into five generations based on the endodomain (Fig. 1).^35–37^

The first-generation CAR-T cells comprised a single fragment derived from the CD3ζ chain.^38–40^ These cells depended on exogenous cytokine production, exhibited insufficient persistence and T cell activation, and consequently, did not achieve the desired results in most studies.^41–45^ Accordingly, first-generation CARs have been superseded by second-generation CARs, which feature an intracellular signaling domain comprising a variety of co-stimulatory protein receptors situated within the cytoplasmic tail of the CARs, such as CD28 or CD137 (4-1BB).^46–49^ These co-stimulatory proteins can enhance proliferation, cytotoxicity, and prolong persistence.

Third-generation CARs integrate multiple signaling domains, including CD28, 4-1BB, ICOS, and/or OX40.^50–52^ Fourth-generation CAR-T cells, also known as “T cells redirected for universal cytokine-mediated killing” or “TRUCKs”^53^ are engineered to release cytokines into the tumor microenvironment (TME). They may also express additional proteins such as chemokine receptors, switch receptors, bispecific T cell engagers (BiTEs), and blockers/inducers of specific signaling pathways.^54–57^

In this context, next-generation CAR-T cells are currently underway. The fifth-generation differs from the previous versions by integrating an additional membrane receptor. Several approaches are being explored, with one of the most promising involving the incorporation of IL-2 receptor signaling to enable antigen-dependent JAK/STAT pathway activation.^58–61^ This signaling not only sustains CAR-T cell activity and promotes memory T cell formation but also reactivates and stimulates the broader immune system.

Modifying T cells to express CARs typically involves transducing the cells with viral vectors containing the transgene, which leads to the semi-random integration of DNA into the T-cell genome. Interestingly, some fifth-generation strategies use specific site-integrations that provide additional features, which can be achieved through CRISPR mediated editing. One example is the insertion of the CAR into the TRAC locus (T cell receptor alpha constant). The TRAC locus is a constant region within the T cell receptor (TCR) alpha chain gene. This genetic editing allows the suppression of the expression of the endogenous TCR to ensure specific antigen recognition while avoiding potential interference from the natural TCR. By integrating TRAC, fifth-generation CAR-T cells maintain greater stability and identity over time, improving their ability to recognize and eliminate cancer cells. This genetic modification reduces the risk of T-cell exhaustion, graft-versus-host effect, and enhances the overall efficacy of CAR-T therapies, offering a more durable and potent treatment option.^62^ In line with this, an additional innovative strategy has integrated the CAR cassette into the *PDCD1* gene locus demonstrating a superior ability to eradicate cancer cells both in vitro and in xenograft models.^63,64^

All six of the currently approved CAR-T cell constructs are second-generation CARs. Axicabtagene ciloleucel (Yescarta®) and brexucabtagene autoleucel (Tecartus®) are CD28-based,^65^ whereas the remaining approved constructs are 4-1BB-based. Most approved products employ a murine scFv except for ciltacabtagene autoleucel (Carvykti®), which utilizes a camelid binding domain.^66^ As exposed in the following sections, to further improve the efficacy of CAR-T cell therapy, the different CAR components have been engineered, resulting in constructs with enhanced properties.^58,67,68^

## CAR-T cell in cancer: current landscape

### History of CAR-T cell implementation

**Fig. 2:**
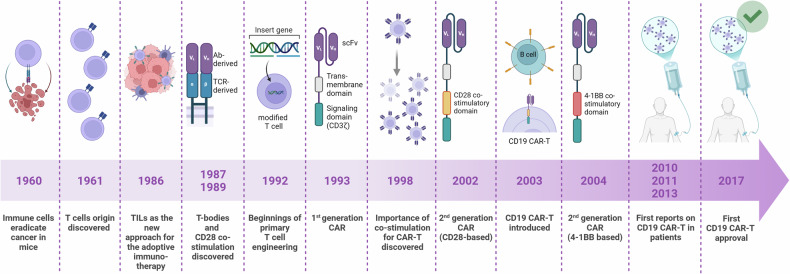
The timeline of milestones in CAR-T cell development. The first reports published in scientific journals or conference abstracts, excluding patent applications, are highlighted. The efficacy of CD19 CAR-T cells was reported in patients with B-NHL in 2010, CLL in 2011, and B-ALL in 2013. The figure was created using Biorender.com

Modern CAR-T cell therapy is the culmination of decades of groundbreaking immunology and genetic engineering research. Here, we provide a brief overview of the key contributions that paved the way for its first approvals and subsequent use in thousands of patients worldwide (Fig. 2).

The remarkable success of CAR-T cell therapy would not have been possible without foundational discoveries in immunology. The identification of T cell origin,^69,70^ and the pioneering work of Dr. Eva and Dr. George Klein, who demonstrated that immune cells can eradicate cancer,^71^ were crucial milestones. In subsequent years, significant contributions were made by Dr. Steven Rosenberg at the National Cancer Institute (NCI), who pioneered the use of tumor-infiltrating lymphocytes (TILs) to treat selected solid tumors in patients.^72–74^ While Rosenberg and colleagues highlighted the undeniable potential of cytotoxic T cells to combat cancer, TIL therapy faced challenges, including low reproducibility in TIL expansion and the restriction of its use to immunogenic tumors due to major histocompatibility complex (MHC) dependency. These limitations spurred researchers to address these challenges and explore ways to harness the anticancer potential of T cells across a broader range of malignancies, including poorly immunogenic tumors. This led to the groundbreaking concept of reprogramming T cells for enhanced specificity.

The concept of CARs originated from two groundbreaking studies conducted in the late 1980s. In 1987, Dr. Yoshikazu Kurosawa and colleagues engineered modified cells expressing T cell receptors (TCRs) with their variable regions replaced by the antigen-binding site of an antibody, creating receptors that functioned independently of MHC interaction.^75^ Similarly, in 1989, Dr. Zelig Eshhar and his team developed analogous constructs.^76^ They successfully generated “T-bodies,” functional chimeric receptors that recognized a hapten, confirmed by their ability to kill target-expressing tumor cells and produce cytokines (Fig. 2). Notably, Eshhar and colleagues proposed for the first time the potential use of CAR-modified T cells to combat cancer, laying the groundwork for subsequent innovations. A few years later, the same team introduced pivotal constructs of what are now considered first-generation CARs (Fig. 1). These early receptors, containing an anti-hapten antibody-derived scFv linked to the intracellular CD3ζ chain, were successfully expressed in murine cytotoxic T lymphocyte (CTL) hybridoma cells.^77^ However, these first-generation CAR-T cells exhibited limited proliferation and killing capacities.^40,44,78^ Moreover, their introduction to murine cells highlighted the need for optimized CAR-T production protocols, particularly to effectively modify and expand human T cells ex vivo. At this time, Dr. Michel Sadelain at Memorial Sloan Kettering Cancer Center (MSKCC) made significant advancements in T-cell engineering by improving retroviral modification methods.^79^ Sadelain’s work on CAR design included the introduction of the CD28 co-stimulatory domain, which enhanced CAR T cell persistence and survival.^80^ These advances were influenced by earlier research led by Dr. Carl June at Children’s Hospital of Philadelphia (CHOP), whose pivotal study a decade earlier established the critical role of CD28 co-stimulation in T cell proliferation and cytokine production, complementing CD3 signaling.^81^ Collectively, the tremendous efforts of scientists in the late 20th century provided the foundational knowledge and technical advancements necessary for the eventual clinical implementation of CAR-T cell therapy.

In the early 2000s, Sadelain’s team emerged as a leader in advancing CAR-T cell research. A pivotal breakthrough came with Maher et al.^82^ who introduced second-generation CARs to primary human T cells (Fig. 1). Although this was not the first report of CARs incorporating the CD28 co-stimulatory domain upstream of the CD3ζ chain,^83^ Maher and colleagues were the first to demonstrate successful modification of primary human T cells to express second-generation CARs targeting prostate-specific membrane antigen (PSMA). These anti-PSMA CAR-T cells exhibited sustained proliferation and responsiveness to repeated stimulation by PSMA-expressing cancer cells, achieving the first fully effective CAR-T product.

Just one year later, researchers from MSKCC delivered another transformative milestone in immunotherapy. While early CAR-T development primarily focused on optimizing signaling constructs to enhance proliferation and persistence, selecting an ideal antigen to target became equally critical. Initial preclinical studies with first- and second-generation CARs targeted solid tumor-associated antigens, including disialogangliosides (GD2, GD3), carcinoembryonic antigen (CEA), and PSMA.^39^ However, Sadelain and his team shifted their focus to hematological malignancies, proposing CD19 as a promising antigen nearly 15 years before its clinical implementation in CAR-T therapy.^84^ In a landmark 2003 study, the MSKCC team demonstrated the feasibility of expanding CAR-T cells ex vivo using artificial antigen-presenting cells and IL-15. These CD19-targeted CAR-T cells achieved sustained persistence in immunodeficient murine models with established B-cell tumors. Around the same time, other research groups in the US—at institutions such as the National Cancer Institute (NCI), CHOP, and Baylor College of Medicine—also identified CD19 as a highly promising tumor-associated antigen.^49,85–87^ These efforts collectively focused on treating B-cell-derived malignancies, including B-cell acute lymphoblastic leukemia (B-ALL), chronic lymphocytic leukemia (CLL), and B-cell non-Hodgkin lymphoma (B-NHL), which uniformly express CD19 on their surfaces (Fig. 2). This concerted effort to target CD19 revolutionized the field and set the stage for CAR-T therapy’s transformative impact on hematologic cancers.

After CD19 was identified as the optimal target for immunotherapy, a critical milestone was the development of scalable manufacturing protocols for clinical-grade CAR-T cells. Until 2010, the efficacy of CD19 CAR-T cells had been primarily evaluated in vitro and in murine models, which required relatively small numbers of effector cells. However, preparing CAR-T cells for clinical use demanded protocols capable of large-scale production. In 2009, Hollyman et al.^88^ described a process for autologous T cell activation, transduction, and expansion in bioreactors, enabling the generation of sufficient cell numbers for clinical applications. This advancement opened new opportunities for future clinical trials. By May 2010, nine independent single-center clinical trials were registered across the US, with three more under regulatory review.^89^ Investigators published data from individual case studies before collective clinical trial results were available. The first report of CD19 CAR-T therapy in a patient came from Rosenberg’s group at the NCI, detailing a case of relapsed B-NHL.^90^ This study used autologous T cells engineered with a second-generation CAR construct incorporating anti-CD19 scFv (anti-FMC63 epitope), a CD28 co-stimulatory domain, and a CD3ζ signaling chain.^90^ In the following months, additional studies reported the use of CD19 CAR-T cells in patients with B-cell-derived malignancies. The first clinical results from the CHOP described treating CLL,^91,92^ while researchers at MSKCC reported outcomes for adult B-ALL.^93,94^ Notably, the CHOP trials utilized CD19 CAR-T cells designed with the 4-1BB co-stimulatory domain (CD19-BB-z), a construct initially introduced by Dr. Dario Campana and Dr. Chihaya Imai at St. Jude Children’s Research Hospital.^49^ These early clinical studies established the foundation for the rapid progression of CAR-T therapy from experimental models to transformative cancer treatments (Fig. 2).

While early CD19 CAR-T cell clinical trials primarily enrolled R/R adult patients, a groundbreaking milestone was achieved in 2012 when physicians and scientists from CHOP administered CD19 CAR-T therapy to the first pediatric patient with B-ALL. The treatment garnered worldwide attention as the patient achieved morphologic remission within a month of CAR-T cell infusion.^95^ Remarkably, as of 2024, this patient remains cancer-free after 12 years, highlighting the life-saving potential of CAR-T therapy.^96^ The treatment, however, was not devoid of high-grade adverse effects and severe cytokine release syndrome (CRS), which was manifested by high IL-6 levels. Fortunately, the CHOP team, led by Dr. Carl June, obtained permission for the “off-label” use of tocilizumab—a humanized monoclonal antibody targeting the IL-6 receptor—originally approved for treating rheumatoid arthritis. The successful management of CRS with tocilizumab was a pivotal moment in CAR-T therapy, and IL-6 receptor antagonists are now the standard of care for CRS management in CAR-T-treated patients,^97,98^ even prophylactically. This case showcased CAR-T cells’ efficacy in pediatric cancer.^99,100^ It underscored the importance of effective strategies to manage treatment-associated toxicities, paving the way for safer and more accessible CAR-T therapies.^100^

In the subsequent years, additional results from single-center trials demonstrated sustained remissions in patients treated with CD19 CAR-T, including adults and children with B-ALL^101,102^ and adults with B-NHL or CLL.^103,104^ The successful responses in these pivotal trials prompted collaborations between scientists, clinicians, and biopharma companies to further develop and implement this treatment, ultimately acquiring the necessary regulatory approvals. Notably, June’s team at CHOP partnered with Novartis,^105^ Rosenberg’s team at NCI collaborated with Kite Pharma,^106^ and researchers from MSKCC, Seattle Children’s Research Institute, and Fred Hutchinson Cancer Research Center founded Juno Therapeutics.^107^ As a result, CD19 CAR-T received breakthrough therapy designation from the FDA in 2014, and the global multi-center trials culminated in the first approvals in 2017. Specifically, Kymriah® (CD19-BB-z) became the first approved cell-based therapy for R/R B-ALL in patients under 25 years old, while Yescarta® (CD19-28-z) was approved for adult R/R B-NHL, based on the ELIANA,^108,109^ and and ZUMA-1^110^ trials, respectively.

These landmark approvals resulted from over 30 years of groundbreaking research and the dedicated efforts of scientists and clinicians. Despite the success of these CAR-T therapies, challenges remain in gaining approval for their use in all blood cancers and solid tumors. In the following sections, we will present an overview of the current role of CAR-T cells in hematologic malignancies with a major focus on AML and solid tumors, followed by the key difficulties involved in implementing adoptive cell therapies for patients with AML.

### CAR-T therapies for hematologic malignancies and solid tumors: current scenario

The US Food and Drug Administration (FDA) has approved six CAR-T cell therapies for the treatment of various hematologic malignancies. Kymriah®, a second-generation CAR-T cell therapy targeting the B cell antigen CD19, was the first CAR-T cell therapy to receive approval by the FDA and the European Medicines Agency (EMA) for the treatment of children and young adults with ALL.^109,111,112^ The FDA and EMA have subsequently approved three additional CAR-T cell therapies targeting the CD19 antigen: Yescarta®,^113–115^ Tecartus®,^65,113^ and lisocabtagene maraleucel (Breyanzi®).^116,117^ In addition, two B cell maturation antigen (BCMA) CAR-T cell products have been approved for the treatment of R/R MM, idecabtagene vicleucel (Abecma®)^118^ in March 2021 and Carvykti® in February 2022.^66,119,120^ Currently, several clinical trials are ongoing testing these six CAR-T cells for further indications (Table 1).

**Table 1:** EMA & FDA approved CAR T cell therapies

Tradename (Generic name)	Target	Approved indication	Manufacturer/marketing authorization issued	Clinical trial	Phase Trial	Aim and participation Criteria	Status	Recruitment	Locations	Efficacy outcomes	Safety outcomes	Ref.
Kymriah(Tisagenlecleucel (tisa-cel)) CTL019	CD19	Adult patients (up to 25 years of age) with Refractory B-cell precursor ALL R/R Large B-cell lymphoma: DLBC, High grade B-cell Lymphoma and DLBCL arising from FL R/R FL	Novartis Pharmaceuticals Corporation EMA (2018) FDA (2017)	NCT04134117	I	To study the safety of tisa-cel in adult patients (≥60 years of age) with R/R Primary CNS Lymphoma	C	9 participants	US	-	**CRS:** 66.67%, 11% grade3 **NE:** 55.6%	^449–451^
				NCT03630159 PORTIA 2018-000973-57 (EudraCT)	I	To evaluate the safety and efficacy of the administration of tisa-cel in combination with pembrolizumab in adult patients with R/R DLBCL, not candidates for ASCT	C	12 participants	US, Austria, Canada	**ORR:** 50% **CR:** 33.3%	**CRS:** 58.3% **ICANS:** 8.3%	^452,453^
				NCT03876028	I	To evaluate the safety and tolerability of the administration of tisa-cel in combination with ibrutinib in adult patients with R/R DLBCL, not candidates for ASCT.	T	10 participants ***Arm 1:*** 4 participants ibrutinib 560 mg/d for ~4 weeks prior to leukapheresis ***Arm 2:*** 6 participants ibrutinib after leukapheresis	US	**CR:** *Arm 1:* 50% *Arm 2:* 33.3%	**CRS: grade 1** *Arm 1: 25*% Arm 2: 83.3% **NE:** Arm 2: 16.7%	^454^
				NCT06003179	I	To identify an optimized lymphodepletion (LD) regimen by evaluating standard and intermediate doses of Fludarabine (Flu)/Cyclophosphamide (Cy) with or without a fixed dose of total lymphoid irradiation (TLI) in the setting of standard of care CAR T cell therapy. ≥18 years Patients	WD	Enrollment estimated 20-40 participants	Canada	-	-	^455^
				NCT05310591 CAPTiRALL	I, II	To determine the safety, efficacy and feasibility of Nivolumab (Opdivo®)- an anti-PD1 treatment- combined to tisa-cel in Patients aged from 1 to 25 years (pediatric and young adults) with a history of CD19 + R/R B-ALL (any relapse after HSCT, 2nd R/R ALL).	R	Enrollment estimated 26 participants	France	-	-	^456^
				NCT03876769 CASSIOPEIA 2017-002116-14 (EudraCT)	II	To determine the efficacy and safety of tisa-cel in de novo High Risk (HR) pediatric and young adult B-ALL patients (1 to 25 years) who received first-line treatment and are MRD Positive at the End of Consolidation (EOC) Therapy	ANR	121 participants	US, Belgium, Canada, Denmark, France, Italy, Netherlands, Norway, Spain, Sweden, UK	-	-	^457,458^
				NCT03568461 ELARA 2017-004385-94 (EudraCT)	II	To determine the efficacy and safety of tisa-cel in adult patients (≥18 years) with R/R FL (Grade 1, 2, 3A)	ANR	***Arm CTL019:*** 98 participants who received tisa-cel infusion	US, Australia, Austria, Belgium, France, Germany, Italy, Japan, Netherlands, Norway, Spain, UK	**ORR:** 86.2% (94 p.a.) **CR:** 69.1% (94 p.a.)	**CRS** 30.93% (97 p.a.) 19.59% serious adverse **ICANS:** 1.03% (97 p.a.)	^111,459^
				NCT04234061 TARMAC	II	To evaluate the efficacy and safety of the combination of ibrutinib and Tisa-cel in ≥18 years of age patients with R/R Mantle Cell Lymphoma (MCL) or who had sub-optimal response to standard therapy in the presence of TP53 mutation.	ANR	20 participants	Australia	-	-	^460^
				NCT02445248 JULIET 2014-003060-20 (EudraCT)	II	To determine the efficacy and safety of (tisa-cel) in adult patients (≥18 years) with histologically confirmed R/R DLBCL after ≥2 lines of chemotherapy, with a life expectancy of ≥12 weeks and not eligible or not consenting to HSCT	C	115 participants: ***Main Cohort (patients treated with tisa-cel US manufacturing facility):*** 99 participants ***Cohort A (patients treated with tisa-cel EU manufacturing facility):*** 16 participants	US, Australia, Austria, Canada, France, Germany, Italy, Japan, Netherlands, Norway	**ORR:** *Main Cohort:* 54.5% Cohort A: 53% **CR:** *Main cohort* 39%	**CRS:** *Main cohort:* any grade 57%, grade 3–4 23% **NE:** **Main cohort:** any grade 20%, grade 3 or worse 11%	^112,461,462^
				NCT03610724 BIANCA 2017-005019-15 (EudraCT)	II	To assess the efficacy and safety of tisa-cel in pediatric, adolescent and young adult patients with R/R B-NHL including Burkitt Lymphoma and Burkitt Leukemia.	C	34 participants (33 infused)	US, Australia, Austria, Canada, Denmark, Finland, France, Germany, Italy, Japan, Netherlands, Norway, Spain, UK	**ORR:** 32.1% (28 p.a.)	**CRS:** 24.24% (33 p.a.) **NE:** 6.06%	^463^
				NCT02228096 ENSIGN 2015-003736-13 (EudraCT)	II	To determine the efficacy and safety of tisa-cel in pediatric patients (3–21 years) with B-ALL, who were refractory to standard chemotherapy regimen or relapsed after HSCT.	C	75 participants	US	**ORR:** 70.3% (64 p.a.) **CR:** 67.2%	**CRS:** 64.06% (64 p.a.) **NE:** 6.25% (64 p.a.)	^464–467^
				NCT05460533	II	To see if early reinfusion of tisa-cel can keep participants in B-cell aplasia at 6 months after their first infusion. To evaluate the safety of early reinfusion and how effective it is at treating B-ALL. Pediatric and Young Adult Patients With R/R CD19-Positive B-Cell ALL (REFUEL)	R	Enrollment estimated 33 patients	US	-	-	^468^
				NCT04225676	II	To determine the efficacy and safety of reinfusion of tisa-cel. Pediatric and adolescent young adult (AYA) patients (≤25 years old) with ALL experiencing loss of B cell aplasia and a previous infusion of commercial tisa-cel with ≥1 additional dose available.	T	5 participants	US	Terminated due to slow enrollment	**CRS:** 20%	^469–471^
				NCT04161118 TIGER-CTL019 Uni-Koeln-3903	II	To determine the efficacy and safety of tisa-cel in elderly Patients (>65 years, or > 60 years old with HCT-CI score >2) with First-Relapsed or Primary Refractory Aggressive B- NHL (treatment with immunochemotherapy containing rituximab and anthracycline)	Termination of the funding agreement by the financial sponsor. Small number of patients enrolled	3 participants	Germany	-	-	^472^
				NCT04156659	II	To evaluate the efficacy and safety of tisa-cel in Chinese Pediatric and Young Adult Patients (age ≤25 years) With R/R B-ALL	WD	Without information	No location data	Not provided	-	^473^
				NCT04456023	II	To evaluate the efficacy and safety of CTL019 in Chinese adult patients (≥18 years of age) with R/R DLBCL.	WD	Without information	Without information	-	-	^474^
				NCT03570892 BELINDA 2016-002966-29 (EudraCT)	III	To compare the efficacy, safety, and tolerability of tisa-cel to SOC in adult patients (≥18 years) with aggressive B-NHL	ANR	***Tisa-cel arm:*** 162 participants ***SOC arm:*** 160 subjects treated with platinum-based immunochemotherapy followed in responding patients by high dose chemotherapy and auto-HSCT	US, Australia, Austria, Belgium, Brazil, China, France, Germany, Hong Kong, Italy, Japan, Netherlands, Norway, Singapore, Spain, Switzerland, Tawian, UK	**ORR:** *Tisa-cel arm:*46.3% *SOC:* 42.5% **CR:** *Tisa-cel arm:* 46% *SOC:* 44%	**CRS:** 61.3% **NE:** 10.3%	^475,476^
				NCT05888493 LEDA 2023-503452-27-00 (Registry Identifier) (REGISTRY: EU CT Number)	III	To compare tisa-cel to SOC in adult patients (≥18 years) with R/R FL after two or more lines of systemic therapy, with progression-free survival (PFS) as the primary endpoint.	R	108 participants ***Arm A:*** tisa-cel treatment ***Arm B:*** SOC. Treatment with R2 or R-CHOP	Australia, Canada, Czechia, Hungary, Republic of Korea, Poland, Romania, Singapore, Slovakia, Spain, Taiwan	-	-	^477^
				NCT04094311	III	To evaluate the safety of tisa-cel that is OOS for release as commercial product. Pediatric/young adult patients with R/R B-ALL and adult patients with R/R LBCL including DLBCL not otherwise specified, high-grade B cell lymphoma, and DLBCL arising from FL for Part 1 and and R/R ALL and R/R NHL for Part 2	R	Enrollment estimated: 200 participants	Canada, Japan	-	-	^478^
				NCT05199961	Not applicable	To evaluate quality of life (QOL) and other patient reported outcomes (PROs) in adults with DLBCL following (CAR) T-cell therapy with tisa-cel	T Sponsor requested early termination	4 participants	US	-	-	^479^
				NCT05541341	Observational	To determine the efficacy and safety of tisa-cel in pediatric (<18 years) and adult patients (aged 18 years or older) with B-cell malignancies who have received tisa-cel through the commercial setting or OOS use in Brazil.	R	Enrollment estimated: 200 participants	Brazil	-	-	^480^
Yescarta (Axicabtagene ciloleucel)	CD19	R/R DLBCL High-Grade B-cell Lymphoma (HGBL) Primary mediastinal large B-cell lymphoma (PMBCL), after two or more lines of systemic therapy. Adult patients with R/R FL after three or more lines of systemic therapy.	Kite Pharma, Inc. Kite Pharma EU B.V EMA (2018) FDA (2017)	NCT03153462 ZUMA-9 2015-005007-86 (EudraCT)	Expanded Access	Expanded access to axi-cel until commercial availability. Age ≥18 years subjects who received an infusion of axi-cel will complete the remainder of the 15-year follow-up assessments in a separate long-term follow-up study, KT-US-982-5968. R/R DLBCL, R/R Primary Mediastinal B Cell Lymphoma,R/R transformed FL, R/R HG B-Cell Lymphoma	AFM	***Cohort 1 (C1):*** 25 participants ***Cohort 2 (C2):*** 36 participants	US	**ORR:** 76% **CR:** 64%	**CRS:** Grade ≥ 3 was not observed in C1. 3% of C2 **NE:** C1: 36% Grade ≥ 3 C2: 19% Grade ≥ 3	^447,481,482^
				NCT04608487	I	To test the safety and effectiveness of axi-cel (axi-cel) to treat CNS Lymphoma. Age ≥18 years participants with R/R active primary or secondary CNS lymphoma (PCNSL or SCNSL), histologically proven aggressive B cell lymphoma, including DLBCL, HGBL, PMBL, or tFL	ANR	18 participants: 13 PCNSL, 4 SCNSL, 1 concurrent systemic & ocular Lymphoma	US	**ORR:** 94% **CR:** 67%	**CRS:** 89%, 0% grade ≥3 **ICANS:** 44%, 28% grade ≥3	^483,484^
				NCT05077527	I	To demonstrate safety and feasibility of axi-cel for R/R human immunodeficiency virus (HIV)-associated aggressive B-NHL in participants with well-controlled HIV	R	20 participants	US	-	-	^485^
				NCT05794958	I	To establish safety of Axi-Cel-2 in patients with LBCL who are at high risk of relapse. Reinfusion (Axi-Cel-2) in patients with R/R second line High-Risk NHL after SOC Axi-Cel	R	20 participants enrollment estimated	US	-	-	^486^
				NCT05950802 ODIN	I	To identify an optimized LD regimen by evaluating standard and intermediate doses of Flu/ Cy with or without a fixed dose of TLI in the setting of standard of care CAR T cell therapy. ≥18 years Patients Receiving CAR T Cell Therapy Axi-cel (ODIN)	R	Enrollment estimated 40 patients	Canada	-	-	^487^
				NCT03704298 ZUMA-11	I	Age ≥18 years participants with R/R LBCL who received an infusion of axi-cel and utomilumab (UT) Phase 1: To evaluate the safety of Axi-cel in combination with UT and to identify the most appropriate dose and timing of UT to carry forward into Phase 2 Phase 2: To evaluate the efficacy of axi-cel and UT as measured by CR.	T Development program T	12 participants: ***Phase 1: Cohort 1:*** Axi-cel + UT 10 mg: 3 participants ***Phase 1: Cohort 2:*** Axi-cel + UT 30 mg+O34:O45: 3 participants ***Phase 1: Cohort 3:*** Axi-cel + UT 100 mg: 3 participants ***Phase 1: Cohort 4:*** Axi-cel + UT 200 mg: 3 participants	US	**ORR:** *Phase 1: Cohort 1:* 67% *Phase 1: Cohort 2:* 33% *Phase 1: Cohort 3:* 100% *Phase 1: Cohort 4:* 100%	**Serious adverse effects:** *Phase 1: Cohort 1:* 33.33% Phase 1: Cohort 2: 100.00% Phase 1: Cohort 3: 33.33% *Phase 1: Cohort 4:* 66.67%	^488^
				NCT04314843 ZUMA-19 2019-004568-23 (EudraCT)	I	Age ≥18 years participants with R/R LBCL ***Phase 1:*** To evaluate the safety of sequenced therapy with lenzilumab and axi-cel ***Phase 2:*** To evaluate the incidence of NE with sequenced therapy given at the recommended Phase 2 dose (RP2D) of lenzilumab	T Development program T and the decision was not due to any safety concerns.	6 patients ***Phase 1 Cohort 1:*** 3 participants ***Phase 1 Cohort 2:*** 3 participants **Phase 2** was not conducted	US	**ORR:** Phase 1 Cohort 1: 67% Phase 1 Cohort 2: 100% **CR:** Phase 1 Cohort 1: 67% Phase 1 Cohort 2: 67%	**CRS:** *Phase 1 Cohort 1: 67%* *Phase 1 Cohort 2:* 67% **NE:** Phase 1 Cohort 1: 100% Phase 1 Cohort 2: 67%	^489–491^
				NCT03105336 ZUMA-5 2017-001912-13 (EudraCT) 2023-505169-10 EMA	II	To evaluate the efficacy and safety of Axi-cel in adult with R/R Indolent NHL (iNHL): patients with FL or Marginal Zone Lymphoma MZL)	ANR	158 participants received lymphodepleting chemotherapy and axi-cel: ***FL:*** 127 ***MZL:*** 31	US, France	**ORR:** *FL:* 94% *MZL:* 77% **CR:** *FL:* 79% *MZL:* 65%	**CRS:**(18-month analysis) *FL:* 0% *MZL:* 3% **NE:**(18-month analysis) *FL:* 0% *MZL:* 3%	^65,492–494^
				NCT05459571 ZUMA-24	II	To learn more about the study drug, axi-cel, in age ≥18 years participants with R/R LBCL in the outpatient setting	ANR	As of November 2, 2023, 23 pts were enrolled and 20 received axi-cel.	US	**ORR:** 90% **CR:** 70%	**CRS:** 85%, 5% Grade ≥3 **NE:** 75%, 25% Grade ≥3	^495–497^
				NCT04002401 ZUMA -14 2019-004803-11 (EudraCT)	II	To estimate the efficacy of axi-cel in combination with rituximab in age ≥18 years participants with R/R LBCL.	C	26 participants	US	**CR:** 65% **ORR:** 88%	**CRS:** There was no Grade ≥3 **NE:** Grade ≥3 15%	^498–500^
				NCT03761056 ZUMA-12 2019-002291-13 (EudraCT)	II	To estimate the efficacy of axi-cel in age ≥18 years participants with HG LBCL	C	40 participants	US, Australia, France	**ORR:** 92% (37 p.a.) **CR:** 86% (37 p.a.)	No new cases of CRS or NE of any grade occurred since the prior data cut.	^501–506^
				NCT06218602	II	To assess the toxicity of combination of FMT with Axi-cel and compare it with SOC Arm B with Axi-cel. Age ≥18 years Lymphoma Patients	R	40 Patients	US	-	-	^507^
				NCT06043323	II	To determine the safety of SOC axi-cel with bridging radiotherapy (RT) in adult patients with R/R FL assessed by the incidence of grade 3 or higher CRS within 30 days after CAR T-cell infusion.	R	20 participants enrollment estimated	US	-	-	^508^
				NCT06213311	II	To evaluate the safety of axi-cel and glofitamab as combination therapy in 2nd line LBCL participants	R	40 participants	US	-	-	^509^
				NCT03391466 ZUMA-7 2017-002261-22 (EudraCT)	III	To assess whether axi-cel therapy improves the clinical outcome compared with SOC second-line therapy in age ≥18 years participants with R/R DLBCL after first-line rituximab and anthracycline-based chemotherapy	ANR	359 participants ***Axi-cel:*** 180 participants ***SOC***: 179 participants	US, Australia, Austria, Belgium, Canada, France, Germany, Israel, Italy, Netherlands, Spain, Sweden, Switzerland, UK	**ORR:** ***Axi-cel:*** 83% ***SOC:*** 50% **CR:** ***Axi-cel:*** 65% ***SOC:*** 32%	**CRS:** *Axi-cel: 92%*, Grade ≥3 6% **NE:** *Axi-cel:* 60% *SOC:* 20%	^510–514^
				NCT05605899 ZUMA-23 2022-501489-24-00 EMA	III	To compare the study drug, axi-cel, versus SOC in first-line therapy in adult participants with high-risk LBCL	R	300 participants enrollment estimated	US, Austria, Canada, Francia, Germany, Italy, Japan, Netherlands, Portugal, Spain, UK	-	-	^515^
				NCT05371093 ZUMA-22 2021-003260-28 EMA 2024-511594-30 EMA	III	To find out how well the drug axi-cel works in patients with R/R FL when compared to SOC. Age ≥18 years participants histologically confirmed FL (Grade 1, 2, or 3a) R/R disease after first-line chemoimmunotherapy and high-risk disease with relapse or progression within 24 months of the initial course of chemoimmunotherapy (ie, POD24), Or R/R disease after ≥2 prior systemic lines of therapy	R	230 participants enrollment estimated	US, France, Germany, Italy, Japan, Spain, UK	-	-	^516,517^
				NCT06609304	IV	To learn if axi-cel works for consolidation after first-line treatment of high-risk LBCL in adult participants untreated CD19-positive LBCL;axi-cel works for consolidation after first-line treatment of high-risk LBCL	R	20 participants enrollment estimated	China	-	-	^518^
				NCT05800067	Not Applicable	To explore the efficacy and safety of Axi-cel retreatment inadult participants with R/R LBCL in Shanghai Ruijin Hospital in China	R	32 participants enrollment estimated	China	-	-	^519^
Tecartus (Brexucabtagene autoleucel) KTE-X19 Brexu-cel	CD19	Adult patients with relapsed or refractory mantle cell lymphoma (MCL) Adult patients with R/R B-cell precursor ALL	Kite Pharmaceuticals, Inc. Kite Pharmaceuticals, Inc.EMA (2020) FDA (2020)	NCT04162756 ZUMA-18	Expanded Access	Cohort 1: to provide access to brexu-cel for individuals R/R MCL until KTE-X19 is commercially available Cohort 2: To provide access to KTE-X19 for individuals with R/R MCL whose commercially manufactured product did not meet commercial release specification(s)	AFM	23 patients Cohort 1: 21 participants Cohort 2: 2 participants	US	**ORR:** 87% **CR:** 57%, 30% had a PR, and 9% had progressive disease (PD)	**CRS: 4%** Grade ≥3 **NE:** 35%	^520,521^
				NCT05776134	Expanded Access	To provide access to brexu-cel for patients diagnosed with a disease approved for treatment with brexu-cel, that is otherwise out of specification for commercial release.	AV	-	US	-	-	^522^
				NCT05993949	I	To assess the feasibility of oral dasatinib pulses (3 consecutive days per week) during the first month following infusion of brexu-cel in adult participants with R/R B-ALL	R	20 participants enrollment estimated	US	-	-	^523^
				NCT03624036 ZUMA-8 2018-001923-38 (EudraCT)	I	To evaluate the safety and tolerability of brexu-cel (KTE-X19) in ≥ 18 years participants with R/R CLL and small lymphocytic lymphoma (R/R SLL) who have received at least 2 prior lines of treatment, one of which must include a Bruton’s tyrosine kinase (BTK) inhibitor.	T Development program T	15 patients received KTE-X19 therapy: Cohort 1: 6 participants R/R CLL Cohort 2: 3 participants R/R CLL Cohort 3: 3 participants R/R CLL and SLL with ≤1% malignant cells in peripheral blood Cohort 4: 3 participants with R/R CLL who previously received two lines of therapy along with ibrutinib with or without anti CD20 antibodies	US, Italy	**ORR:** Cohort 1: 50% Cohort 2: 33% Cohort 3: 100% Cohort 4: 0%	**CRS** 80%, 7% grade ≥3 **NE:** 73%, 20% grade ≥3	^524–526^
				NCT02625480 ZUMA-4 2015-005010-30 (EudraCT)	I II	To evaluate the safety and efficacy of (KTE-X19) in pediatric and adolescent participants with R/R B-precursor ALL or R/R (NHL). Up to 21 years pediatric and adolescent participants	ANR	24 patients	US, Canada, Czechia, France, Germany, Italy, Netherlands, Poland, Spain, Sweden	**ORR:** 67% **CR:** 29%, 38% CRi	**CRS:** 88%, 33% grade ≥3 **NE:** 67%, 21% grade ≥3	^527,528^
				NCT02614066 ZUMA-3 2015-005009-35 (EudraCT)	I II	To determine the safety and efficacy of (KTE-X19) in ≥ 18 years participants with R/R B-precursor ALL	C	100 patients ***Phase 1:*** 45 patients ***Phase 2:*** 55	US, Canada, France, Germany, Netherlands	**ORR:** *Phase 1:* 69% **CR:** *Phase 1:* 53%, 16% achieving CRi *Phase 2:* 56.4%	**CRS:** Phase 1: 93%, 31% grade ≥3 Phase 2: 24% grade ≥3 **NE:** Phase 1: 78%, 38% grade ≥3 Phase 2: 25%	^529–531^
				NCT06287229 NCI-2024-01756 (OTHER: NCI-CTRP Clinical Registry)	I II	To learn about the safety of giving the drug brexu-cel to adult participants with R/R B-ALL after treatment with inotuzumab ozogamicin, blinatumomab, and either hyper-CVAD or mini-hyper-CVD	R	40 participants enrollment estimated	US	-	-	^532^
				NCT05537766 ZUMA-25 2022-501259-10 EMA	II	To test how well the study drug, brexu-cel, works in participants with rare B-cell malignancies. ≥18 years participants with R/R Waldenstrom macroglobulinemia (R/R WM) (Substudy A - no longer recruiting), R/R Richter transformation (R/R RT) (Substudy B), R/R Burkitt lymphoma (R/R BL) (Substudy C and/r hairy cell leukemia (R/R HCL) (Substudy D - no longer recruiting).	ANR	19 participants	US, Austria, France, Germany, Italy, Netherlands, Spain, Switzerland	-	-	^533^
				NCT02601313 ZUMA-2 2015-005008-27 (EudraCT) 2023-506641-35 EMA	II	To test how well the study drug, KTE-X19, works in adult participants with R/R MCL who have been treated with up to 5 prior regimens including a Bruton’s tyrosine kinase inhibitor (BTKi) in Cohort 1 and 2	C	92 patients Cohort 1: 68 participants treated with 2 ×10^6 Brexu-cel Cohort 2: 14 participants treated with 0.5 ×10^8 Brexu-cel 2 ×10^6 Axi-cel: 10 participants	US, France, Germany, Netherlands	**ORR:** Cohort 1:93% cohort 2: 93%	**CRS:** 15% grade 3 or higher **NE:** 31%	^534–536^
				NCT06553872	II	To study the combination of two treatments, Pirtobrutinib and brexu-cel, for patients with R/R MCL Age ≥ 18 years participants with R/R MCL who meet the criteria for standard-of-care FDA label for CD19 CAR T-cell therapy with brexu-cel	R	60 participants enrollment estimated	US	-	-	^537^
				NCT04880434 ZUMA-2 2015-005008-27 (EudraCT)	III	To test how well the study drug, brexu-cel (KTE-X19), works in adult participants with R/R MCL up to 5 prior regimens for MCL	ANR	86 participants	US, France, Germany, Netherlands, Spain, UK	**ORR:** 91% **CR:** 73%	**CRS:** 6% **ICANS:**21% **NE:** 27%	^538,539^
Abecma (idecabtagene vicleucel) bb2121 ide-cel	BCMA	R/R MM	Celgene Corporation, a Bristol-Myers Squibb Company Celgene Corporation, a Bristol-Myers Squibb Company Celgene Corporation, a Bristol-Myers Squibb Company EMA (2021) FDA (2021)	NCT03274219	I	To study the dose escalation trial of bb21217 in adult R/R MM patients who received ≥3 prior regimens, including proteasome inhibitor and immunomodulatory agent, or are double refractory to both classes.	C	As of March 1, 2020, 46 participants: 24 participants in escalation: 12 at 150, 6 at 300 and 6 at 450 22 in expansion: 8 at 300 and 14 at 450	US	**CR:** 55%	**CRS:** 67% **NE:** 22%	^540,541^
				NCT04771078	Expanded Access	To evaluate the safety and efficacy of nonconforming ide-cel in adult participants with MM per the approved prescribing information.	AV	31 participants 8 participants received LEN maintenance after ide-cel at the investigator’s discretion	US	**ORR:** 87.1% *ORR LEN maintenance:* 100% **CRR:** 80.6% CR *LEN maintenance:* 75%	-	^542,543^
				NCT06048250	I	To study the safety, side effects, best dose and effectiveness of mezigdomide (CC-92480) when given after ide-cel in adult patients with MM that has come back after a period of improvement (relapsed)	R	15 participants enrollment estimated	US	-	-	^544^
				NCT04855136 KarMMa-7 2020-003248-10 (EudraCT)	I II	To determine the safety, tolerability, efficacy, PK of bb2121 in combination with other therapies in adult subjects with R/R MM.	ANR	128 participants Arm A: bb2121 in combination with CC-220 ( ± low-dose dexamethasone) Arm B: bb2121 in combination with BMS-986405 (JSMD194)	US, Spain	**ORR:** 73% **CR:** 33%	**CRS:** 84% **NE:** 18%, 3% had grade 3 and 0 had grade ≥ 4	^545–547^
				NCT06518551	I II	To evaluate the efficacy of Elotuzumab and Iberdomide therapy post-Ide-cel in adult participants with R/R MM	R	49 participants enrollment estimated	US	-	-	^548^
				NCT05032820 BMTCTN1902	II	To assess bb2121 to improve post AHCT responses among adult patients with MM and sub-optimal response after AHCT and maintenance Lenalidomide	ANR	40 participants	US	-	-	^549^
				NCT06523621	II	To evaluate if treatment with adjuvant nivolumab improves depth of response in adult patients withR/R MM who achieve a less-than-ideal response to ide-cel.	NYR	50 participants enrollment estimated	US	-	-	^550^
				NCT05393804	II	To see if the quality of T cells used to create ide-cel (bb2121) affects how ide-cel prevents cancer from coming back in adult patients with R/R MM, and who have had an HCT	R	32 participants enrollment estimated	US	-	-	^551^
				NCT06179888	II	To compare iberdomide maintenance therapy to disease monitoring for improving survival in adult patients who have received ide-cel for MM	R	78 participants enrollment estimated	US	-	-	^552^
				NCT06045806 KarMMa-9 2022-501346-30 (EudraCT)	III	To compare the efficacy, safety, and tolerability of ide-cel with lenalidomide (LEN) maintenance to that of LEN maintenance alone in adult participants with Newly Diagnosed MM (NDMM) who have achieved a suboptimal response post auto-HSCT	ANR	618 participants enrollment estimated	United States, Australia, Austria, Belgium, Canada, Czechia, Denmark, France, Germany, Greece, Israel, Italy, Japan, Republic of Korea, Norway, Poland, Romania, Spain, UK	-	-	^553,554^
				NCT06698887	Observational	To monitor the long-term safety of adult participants who received ide-cel treatment as part of the KarMMa-9 (CA089-1043) Phase 3 clinical trial.	R	15 participants enrollment estimated	Republic of Korea	-	-	^555^
Breyanzi (Lisocabtagene maraleucel) JCAR017	CD19	Adult patients with R/R LBCL, including: DLBCL not otherwise specified (including DLBCL arising from indolent lymphoma), high-grade B cell lymphoma, primary mediastinal LBCL, and FL grade 3B	Juno Therapeutics, Inc., a Bristol-Myers Squibb EMA (2022) FDA (2021)	NCT02631044 TRANSCEND-NHL-001	I	To evaluate the safety, PK, and antitumor activity of modified T cells (JCAR017) administered to adult patients with R/R B-cell NHL PMBCL, FL grade 3B, and MCL. This study will evaluate and refine the dose and schedule of JCAR017 to optimize safety and antitumor activity.	C	At data cutoff (01/19/2023): 88 participants received liso-cel Efficacy set (n = 83)	US	**ORR:** 83.1% **CR:** 72.3%	**CRS:** 61% **NE:** 31%	^117,556–560^
				NCT05075603	I	To evaluate the safety, tolerability, and preliminary anti-tumor activity of NT-I7 administration following standard of care CD19 CAR T-cell therapy for eligible subjects with R/R LBCL.	R	57 participants enrollment estimated	US	-	-	^561^
				NCT03331198 TRANSCEND-CLL-004	I II	To determine the efficacy and safety of JCAR017 in adult subjects with relapsed or refractory CLL or SLL	R	117 patients received liso-cel	US, Canada	**ORR:** 44%	**CRS:** 85%, 8% grade >3 **NE:** 45%, 11% experienced a grade 1, 15% had a grade 2, 18% had a grade 3, and 1% had a grade 4	^562–564^
				NCT03743246 2018-001246-34 (EudraCT)	I II	To evaluate the safety and efficacy of JCAR017 in pediatric subjects aged ≤ 25 years with CD19 + R/R B-ALL and B-NHL	T (Absence of significant therapeutic benefit over existing therapies)	21 participants A. 0.05 ×10^6 CAR + T Cells/kg 9 B. 0.15 ×10^6 CAR + T Cells/kg 8 C. 0.50 ×106 CAR + T Cells/kg 3 D. Not Assigned 1	US, France, Germany, Italy, Netherlands, Spain	**ORR** A: 50% B: 25% C:100%	**CRS** A: 0.00% B: 50.00% C: 0.00% D: 0.00% **Neurotoxicity** A: 14.29% B: 16.67% C: 100.00% D: 0.00%	^565^
				NCT03483103 TRANSCEND-PILOT-017006	II	To determine the efficacy and safety of liso-cel in adult subjects who have R/R B-cell NHL and are ineligible for HSCT	C	61 received liso-cel (efficacy and safety sets)	US	**ORR:** 80% **CR:** 53%	**CRS:** 38% **NE:** 31%	^116,566–568^
				NCT03484702 TRANSCENDWORLD 2017-000106-38 (EudraCT)	II	To evaluate the efficacy and safety of JCAR017 in adult participants with aggressive B-NHL	C	113 patients ***Cohort 1 C1:*** Diffuse B-cell Lymphoma Who Failed ≥ 2 Lines of Therapy: 45 ***Cohort 2 C2:*** Transplant Ineligible Diffuse B-cell Lymphoma Who Failed First Line Therapy: 32 ***Cohort 3 C3:*** Japan Specific - Meeting Eligibility Criteria for Cohort 1 or 2: 14 ***Cohort 4 C4:*** Newly Diagnosed HGBL:4 ***Cohort 5 C5:*** Primary Central Nervous System Lymphoma:7 ***Cohort 7 C7:*** Meeting Cohort 1 Criteria Suitable for Treatment in an Outpatient Setting:11	Austria, Belgium, Finland, France, Germany, Italy, Japan, Netherlands, Spain, Switzerland, UK	**ORR:** *C1:* 61.1% 36 patients analyzed (p.a) *C2:* 63% 27 p.a. *C3:* 70% 10 p.a. *C4:* 100% 1 p.a. *C5:* 80% 5 p.a. *C7:* 88.9% 9 p.a. **CR:** *C1:* 33.3% 36 p.a. *C2:* 48.1% 27 p.a. *C3:* 50% 10 p.a. *C4:* 0% 1 p.a. *C5:* 0% 5 p.a. *C7:* 88.9% 9 p.a.	**CRS:** *C1:* 15.56% 45 p.a. *C2:* 3.13% 32 p.a. *C3:* 0% 14 p.a. *C4:* 0% 4 p.a. *C5:* 0% 7 p.a. *C7:* 0% 11 p.a.	^567,569^
				NCT03744676 TRANSCEND-OUTREACH-007	II	To determine the safety, PK, and efficacy of liso-cel (JCAR017) in adult patients who have R/R for aggressive B-NHL in the outpatient setting.	C	104 patients	US	***ORR:*** 80.5% 82 p.a. ***CRR:*** 53.7% 82 p.a.	**CRS: 0% g**rade ≥ 3 (82 p.a.) **NT:** 9.8% grade ≥ 3 (82 p.a.)	^570–572^
				NCT05583149	II	To assess the effectiveness and safety of acalabrutinib combined with liso-cel for adult patients with R/R aggressive B-cell lymphoma	R	27 participants enrollment estimated	US	-	-	^573^
				NCT05672173	II	To test how well adding liso-cel to nivolumab and ibrutinib works in treating adult patients with Richter’s transformation	R	20 participants enrollment estimated	US	-	-	^574^
				NCT05873712	II	To evaluate the efficacy of the combination of zanubrutinib and liso-cel for the treatment of Richter’s syndrome	R	24 participants enrollment estimated	US	-	-	^575^
				NCT04245839 TRANSCEND FL 2024-510966-18 EU CTR)	II	To evaluate efficacy and safety of JCAR017 in adult subjects with R/R FL or MZL	R	276 participants enrollment estimated	US, Austria, Canada, France, Germany, Italy, Japan, Spain, Sweden, UK	-	-	^576^
				NCT03575351 TRANSFORM 2018-000929-32 (EudraCT)	III	To compare safety and efficacy between the SOC strategy versus JCAR017 in adult participants with R/R NHL	C	184 participants ***SOC Arm:*** 92 participants ***Liso-cel Arm:*** 92 participants	US, Belgium, Finland, France, Italy, Japan, Netherlands, Spain, Sweeden, Switzerland, UK	**ORR:** *SOC Arm: 48.9% 92 p.a*. *Liso-cel Arm: 87% 92 patients p.a*. **CRR:** *SOC Arm:* 43.5% 92 p.a. *Liso-cel Arm:* 73.9% 92 p.a.	**CRS:** SOC Arm: 0.00% only 0/30 Liso-cel Arm: 13.04% only 12/92 **NE:** 11%	^557,577–579^
				NCT06313996 2023-507477-18 EudraCT	III	To evaluate the efficacy and safety of Liso-cel compared to SOC in adults with R/R FL	NYR	300 participants enrollment estimated	No location data	-	-	^580^
				NCT06205290	III	To compare the efficacy and safety of liso-cel vs Investigator’s Choice options (idelalisib + rituximab or bendamustine + rituximab) in adult participants with R/R CLL or SLL, whose disease has failed treatment with both BTKi and BCL2i targeted therapies.	WD	200 participants enrollment estimated	US, Austria, Belgium, France, Germany, Italy, Netherlands, Norway, Spain, Sweden, UK	-	-	^581^
Carvykti (ciltacabtagene autoleucel) JNJ-68284528 LCAR-B38M	BCMA	Patients with R/R MM who have received at least one prior line of therapy, including a proteasome inhibitor and an immunomodulatory agent, and are refractory to lenalidomide	Janssen Biotech, Inc. EMA (2022) FDA (2022)	NCT06623630	I	To evaluate low-dose total body irradiation (TBI) in combination with Cy as lymphodepletion prior to administration of cilta-cel will be safe and tolerable in patients with MM who have impaired renal function To evaluate low-dose TBI-Cy as lymphodepletion prior to cilta-cel will result in comparable CAR T expansion/persistence and disease response rates as those seen with standard lymphodepleting chemotherapy (flu/cy)	R	12 participants enrollment estimated	US	-	-	^582^
				NCT03090659 LCAR-B38M LEGEND-2	I II	To determine the safety and efficacy of LCAR-B38M CAR-T cells in treating adult patients diagnosed with R/R MM	ANR	74 participants	China	**ORR:** 87.8% **CR:** 73.0%	**CRS:** 91.9%, 9.5% had grade ≥ 3 **NE:** 1.3%	^583–587^
				NCT03548207 CARTITUDE-1	I II	To characterize safety of JNJ-68284528 and establish the recommended Phase 2 dose (RP2D) (Phase 1b) and to evaluate the efficacy of JNJ-68284528 (Phase 2). Adult participants with MM The dose selected at the completion of phase 1b will be used in Phase 2.	C	106 participants ***Phase 1b:*** (US Population) 29 ***Phase 2:*** (US Population) 68 ***Phase 2:*** (Japan Population) 9	United Staes, Japan	**ORR:** *Phase 1b:* 29 *Phase 2 US:* 97.1 68 p.a. *Phase 2 Japan:* 100% 8 p.a.	**CRS:** *Phase 1b*: 17.24% *Phase 2 US:* 22.06% *Phase 2: Japan* 11.11% **Neurotoxicity:** *Phase 1b :* 0.00% *Phase 2 US:* 2.94% *Phase 2 Japan:* 0.00%	.^120,588,589^
				NCT05347485	II	To evaluate the efficacy and safety of cilta-cel OOS Age ≥ 18 years participant is suffering from serious or life-threatening MM per USPI (or locally approved label, respectively), and re-apheresis, re-manufacturing, or other anti-myeloma directed therapies is not considered feasible or adequate per investigator	C	86 participants	US	-	-	^590^
				NCT03758417 CARTIFAN-1	II	To evaluate the efficacy and safety of LCAR-B38M chimeric antigen receptor T (CAR-T) cells. In adult chinese participants with R/R MM	R	48 patients received a cilta-cel infusion	China	**ORR:** 89.6% **CR:** 77.1%	**CRS:** 97.9%, 35.4% grade 3/4	^591,592^
				NCT04133636 2018-004124-10 (EudraCT) CARTITUDE-2	II	To evaluate the overall MRD negative rate of participants who receive JNJ-68284528. ≥ 18 years participants with R/R MM	R	As of the 8 October 2021 data cutoff 20 were treated with cilta-cel.	US, Belgium, France, Germany, Israel, Netherlands, Spain	**ORR:** 60.0% **CR:** 30%	**CRS:** 60% **NE:** 20%	^593,594^
				NCT06550895	II	To define the safety of Cilta-cel and Talquetamab in participants with high-risk MM	R	30 participants enrollment estimated	US, Australia	-	-	^595^
				NCT06574126 CAR-HiRiSMM	II	i) to evaluate the proportion of high-risk SMM adult patients with undetectable MRD at 6, 12, and thereafter every 12 months up to 5 years after cilta-cel administration ii) to annotate frequency and severity of adverse events (SAE)	R	20 participants enrollment estimated	Spain	-	-	^596^
				NCT06577025 aMMbition 2023-505792-71-00 EUCT)	II	To evaluate the rate of response (how effectively treatment is working) with signs of potential cure at 5 years after the start of induction treatment. 18 to 70 years participants with documented new diagnosis of MM	R	40 participants enrollment estimated	US, Australia, Brazil, Spain	-	-	^597^
				NCT04181827 CARTITUDE-4 2019-001413-16 (EudraCT)	III	To compare the efficacy of JNJ-68284528 cilta-cel with SOC, either Pomalidomide, Bortezomib and Dexamethasone (PVd) or Daratumumab, Pomalidomide and Dexamethasone (DPd). Age ≥18 years participants with R/R MM	ANR	419 participants: ***Cilta-cel:*** 208 participants ***SOC:*** 211 participants	US, Astralia, Belgium, Denmark, France, Germany, Greece, Israel, Italy, Japan, Republic of Korea, Netherlands, Poland, Spain, Sweden, UK	**ORR:** *Cilta-cel:* 84.6% *SOC:* 67.3% **CR:** Cilta-cel: 73.1% SOC: 21.8%	**CRS:** 76.1, 1.1% grade 3 or 4 **ICANS:** 4.5%	^66,598^
				NCT04923893 2021-001242-35 (EudraCT)	III	To compare the efficacy of Bortezomib, Lenalidomide and Dexamethasone (VRd) induction followed by a single administration of cilta-cel versus VRd induction followed by Lenalidomide and Dexamethasone (Rd) maintenance in newly diagnosed MM participants for whom ASCT is not planned as initial therapy in terms of Progression Free Survival (PFS).	ANR	743 participants	US, Australia, Austria, Belgium, Brazil, Canada, Czechia, Denmark, Finland, France, Germany, Greece, Hungary, Israel, Japan, Republic of Korea, Netherlands Norway, Poland, Portugal, Spain, Sweden, Switzerland, UK	-	-	^599,600^
				NCT05257083 2021-003284-10 (EudraCT)	III	To compare the efficacy of Daratumumab, Bortezomib, Lenalidomide and Dexamethasone (DVRd) followed by Cilta-cel versus Daratumumab, Bortezomib, Lenalidomide and Dexamethasone (DVRd) followed by ASCT in newly diagnosed MM patients.	R	750 participants enrollment estimated	US, Australia, Belgium, Canada, Czechia, France, Germany, Greece, Israel, Italy, Japan, Netherlands, Norway, Spain, Sweden, Switzerland, UK	-	-	^601^
				NCT05201781 2020-005521-84 (EudraCT)	IV	To collect long-term follow-up data on delayed adverse events after administration of cilta-cel, and to characterize and understand the long-term safety profile of cilta-cel.	R	228 participants enrollment estimated	United Stated, Belgium, China, France, Israel, Japan, Netherlands, Spain	-	-	^602^

Data from https://clinicaltrials.gov as of December 28 2024. ANR active not recruiting. AV Available. C Completed, CNS central nervous system, CR Complete Response, CRS Cytokine Release Syndrome, DLBCL Diffuse Large B-cell Lymphoma, FL Follicular Lymphoma, ICANS Immune effector Cell-Associated Neurotoxicity Syndrome, LBCL Large B-cell Lymphoma, NE Neurologic Events, NYR Not yet Recruiting, ORR Overall Response Rate, OOS Out Of Specification, P,a, participants analyzed, R Recruiting, R/R Relapse/Refractory, SOC Standard Of Care, T Terminated, UN Unknown, WD Withdrawn

CAR-T therapy has been a breakthrough treatment for hematologic cancers, but its effectiveness in solid malignancies has been limited. To date, no CAR-T cell therapy has been granted FDA or EMA approval for solid tumors (Table 1), highlighting a critical need for progress in this context. Several factors may contribute to the limited effectiveness of CAR-T cells in solid malignancies, such as the antigen heterogeneity and the tumor microenvironment (TME). The TME is characterized for being highly immunosuppressive, hypoxic, and fibrotic, thus creating a physical and biological barrier that prevent CAR-T cells from accessing the tumor cells. Moreover, several studies have shown limited CAR-T cell expansion and shorter persistence in patients with solid tumors.^121–123^

A clinical case report indicates a response to multiple doses of intracranial IL-13Ra-targeted CAR therapy in a patient with recurrent multifocal glioblastoma, which was sustained for 7.5 months after starting treatment.^124^ Some evidence regarding GD2-specific CAR-T cells in four pediatric patients with H3K27M-mutated glioma has also been reported, with three of four patients exhibiting clinical and radiographic improvement.^125^

Recent studies with larger cohorts in the solid tumor field have demonstrated significant antitumor effects. Notably, a claudin18.2-targeted CAR has proven effective in treating gastrointestinal tumors in a study including 37 patients (NCT04196413), with an overall response rate (ORR) and disease control rate (DCR) of 48.6% and 73.0%, respectively.^126^ Moreover, a GD2-specific CAR has shown strong results in 27 children with heavily pretreated neuroblastoma (NCT03373097). In this study, ORR was 63%; 9 patients had a complete response, and 8 had a partial response. Among those patients who received the recommended dose, the 3-year overall survival and event-free survival were 60% and 36%, respectively. The security profile was reasonable.^127^

While CAR-T cell therapies are not yet approved for solid tumors, other T lymphocyte-based treatments have recently been authorized. One of them is the gp100 peptide-MHC/CD3 bispecific T-cell engager (TCE) tebentafusp which was approved for uveal melanoma in 2022.^128^ Moreover, an autologous TCR T cell therapy named afami-cel is currently being tested in heavily pre-treated patients with HLA-A*02 and MAGE-A4-expressing synovial sarcoma. The phase 2 trial (NCT04044768) resulted in an ORR of 37% and durable responses.^129^ Other cellular therapies have also shown promising responses in treating certain solid tumors like HPV-associated cancers.^130–132^

Collectively our understanding of the underlying reasons for the humble clinical activity observed in CAR-T cell trials involving solid tumors remains under investigation. In the following pages, we will examine the current application of CAR-T cells in the treatment of another disease, which, similar to solid tumors, remains challenging for CAR-T cell therapy: acute myeloid leukemia (AML).

## CAR-T cell-associated challenges in AML

**Fig. 3:**
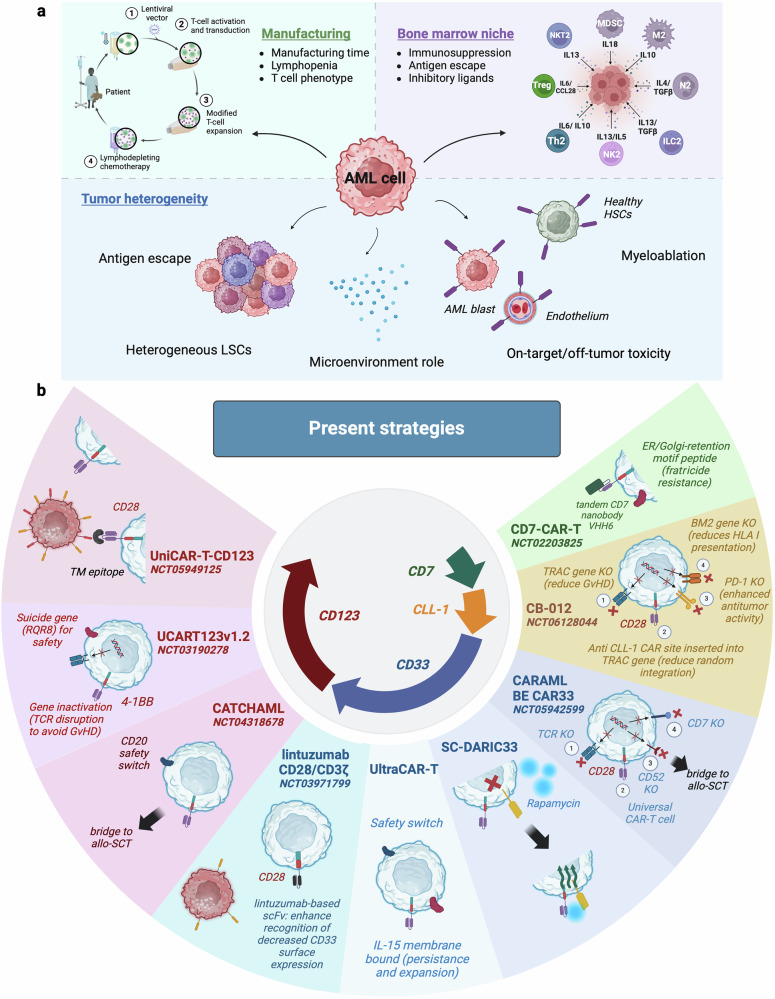
Challenges in CAR-T cell generation for AML: manufacturing, bone marrow niche and tumor heterogeneity role (**a**). Current strategies being tested in clinical trials targeting CD123, CD33, CLL-1 and CD7 antigens (**b**). This figure was created with Biorender.com

The experience gained from treating R/R B-cell malignancies and R/R MM with CAR-T cell therapy has identified several characteristics associated with promising outcomes. These include the CAR molecular structure and co-stimulatory domains, the targeted antigen, the method of transduction, the lymphodepletion regimen prior to cell infusion, the infused cell doses, the heterogeneity of the patient population, and the intrinsic features of the tumor cells, among others. The primary challenges in the AML setting are three-fold: i) the clonal heterogeneity of the disease, ii) the highly immunosuppressive bone marrow (BM) microenvironment, and iii) the lack of tumor-specific target antigens (Fig. 3).^22,133,134^

### AML heterogeneity

Nowadays, acute myeloid leukemia (AML) is a broad category that encompasses various diseases, each with distinct molecular and cytogenetic abnormalities.^135–137^ This molecular and cytogenetic heterogeneity is reflected in the current diagnostic reality, where there are three major international classifications: the WHO 2022,^138^ which defines 11 AML groups based on genetic abnormalities; the ELN 2022,^139^ which is based on the previous one, but identifies 14 AML groups and is focused on prognosis and management; and the ICC 2022,^140^ which recognizes 18 entities. While new risk-stratifying molecular subgroups of AML may emerge over time, not all gene expression subtypes correlate well with disease-associated gene fusions or mutations.^141^

This heterogeneity arises from multiple factors influencing disease presentation, progression, and response to treatment. AML is characterized by a diverse array of genetic mutations that affect key pathways, including signal transduction (*FLT3*), epigenetic regulation (*DNMT3A, IDH1/2, EZH2*), and apoptosis (*TP53*).^4^ These mutations drive distinct transcriptional programs, contributing to variability in disease behavior and drug sensitivity.^142^ Additionally, chromosomal translocations (e.g., *t*(8;21), inv(16), *t*(15;17)) further influence AML prognosis and treatment strategies. Another layer of complexity comes from the clonal evolution of AML, where subclones with distinct genetic profiles contribute to intratumoral heterogeneity.^143^ These molecular and cytogenetic alterations serve as the foundation for prognostic classifications, as they are crucial factors influencing treatment outcomes and survival. For instance, AML with a complex or monosomal karyotype, structural abnormalities involving chromosome 3, *TP53* mutations, high allelic ratio *FLT3* mutations, or mutations in *ASXL1, BCOR, EZH2, RUNX1, SF3B1, SRSF2, STAG2, U2AF1*, and *ZRSR2* (classified as AML with myelodysplasia-related gene mutations),^140^ are associated with treatment resistance and relapse, placing it in the ELN 2022 adverse prognostic group.^139^

AML is thought to mirror normal hematopoiesis, with leukemia stem cells (LSCs) sustaining the disease by driving the production of differentiated blasts.^144^ First described in the 1960s, LSCs are characterized by low cycling or quiescence, self-renewal capacity, and transcriptional or epigenetic signatures similar to hematopoietic stem cells (HSCs) and normal multipotent hematopoietic progenitors. LSCs exhibit therapy resistance due to their quiescent state and self-renewal potential.^145^ They originate from the sequential accumulation of somatic mutations in HSCs or HSPCs, or even more differentiated cells, where early mutations enhance self-renewal and impair differentiation, leading to the expansion of pre-leukemic clones. Common early mutations involve genes regulating epigenetics (*DNMT3A, TET2, IDH1/2, ASXL1*) and *TP53*, while later mutations, such as those in *FLT3*, drive proliferation, block differentiation, and ultimately lead to AML blast formation.^146^

This developmental hierarchy is even more complex at relapse, comprising distinct subclones of leukemia cells with disparate phenotypic, genetic and epigenetic features coexisting within a single patient.^4,141,147^ This complexity may impede CAR-T cell development by facilitating antigen escape.^137^

### BM microenvironment

AML blasts and LSCs present immune evasion mechanisms, in addition to remodeling of the BM microenvironment, which collectively drives disease progression.^19,148^ While these factors were once considered to have a limited role, recent studies have highlighted their crucial contribution to the advancement of the disease.

First, among the intrinsic factors directly related with the myeloid blast, mutations in *NPM1* and *FLT3* have been linked to alterations in immune response.^137,149^ Furthermore, the effects of mutant *IDH1/2* and the subsequent increase of the oncometabolite R-2-hydroxyglutarate (R2-HG) have been associated with the augment of regulatory T cells (Tregs) in AML.^150,151^ *TP53* mutations have also been shown to impede immune surveillance.^152–154^ Other intrinsic features described in AML include downregulation of HLA molecules, leading to defective antigen presentation^155–157^; and alterations in cytokine balance.^158–160^

Secondly, it is well documented that the highly immunosuppressive BM microenvironment deleteriously affects immune responses and T cell fitness.^161^ On one hand, metabolic products within the TME play a significant role in immune suppression, limiting the effectiveness of immunotherapies. AML cells exhibit altered metabolism, producing lactate, adenosine, and kynurenine, which contribute to a hostile microenvironment that impedes the function of CAR-T cells.^162–164^ Elevated lactate levels, resulting from rapid glycolysis in AML blasts, can acidify the TME, suppressing T cell activation and proliferation. This acidic environment not only limits the effector functions of CAR-T cells but also promotes the accumulation of Tregs and myeloid-derived suppressor cells (MDSCs, pathologically activated neutrophils and monocytes with potent immunosuppressive activity^165–167^), both of which further dampen immune responses.^133^ Additionally, adenosine, often elevated in the TME, binds to receptors on CAR-T cells, leading to immune suppression and exhaustion.^168,169^ Similarly, kynurenine, generated through tryptophan catabolism by AML cells, also inhibits T cell function and promotes an immunosuppressive setting.^170^

On the other hand, AML is characterized by an increase in the number of T cells that infiltrate the BM compared to those observed in healthy individuals.^171^ These T cells exhibit an increase in the frequency of immune inhibitory and activating co-receptor expression, particularly in R/R AML.^172–175^ This includes the expression of PD-1,^176,177^ OX40,^178^ TIM3, and LAG3.^179^ Overall, T cells display insufficient potency, persistence, and functionality in this context.^180^ This T cell exhaustion, which may be present in the apheresis-derived T cells or emerge during the CAR-T cell manufacturing process,^181,182^ is typically not only characterized by high expression of inhibitory receptors,^183^ but also is related to extensive transcriptional and epigenetic alterations, defective cytokine production, increased chemokine expression, and a shift from T cell to NK-like T cell phenotypes.^175,184,185^

In third place, in addition to intrinsic factors directly associated with the myeloid blast, its metabolism and other factors related to T cells, the expansion of M2 macrophages has also been shown to enhance AML immune escape.^186,187^ Moreover, poor immune synapse function^187^ and impaired NK cell function have been identified as mechanisms involved in this process.^188–190^ Finally, it is also important to consider the role that the bone marrow structure may play beyond the cellular components. BM vascular remodeling may hinder anti-AML immune responses by reducing cell migration and inducing hypoxia.^191,192^

### Target antigen

The identification of an appropriate target for CAR-T cell therapy in AML is crucial. A suitable target should be an HLA-unrestricted antigen/protein expressed on the cell surface of malignant cells with a high expression level. Nonetheless, some preliminary evidence suggests that lower expression levels might be sufficient to direct CAR-T cells.^22^ Ideally, the antigen should also be expressed on LSCs to ensure complete disease eradication and minimize early relapses.^179^ Importantly, it must be absent in healthy tissues to prevent potentially fatal on-target/off-tumor toxicities.^193^ To date, no optimal target for CAR-T cell therapy in AML has been identified. AML neoantigens are relatively uncommon and are known to be mainly intracellular, requiring HLA presentation.^194^

**Fig. 4:**
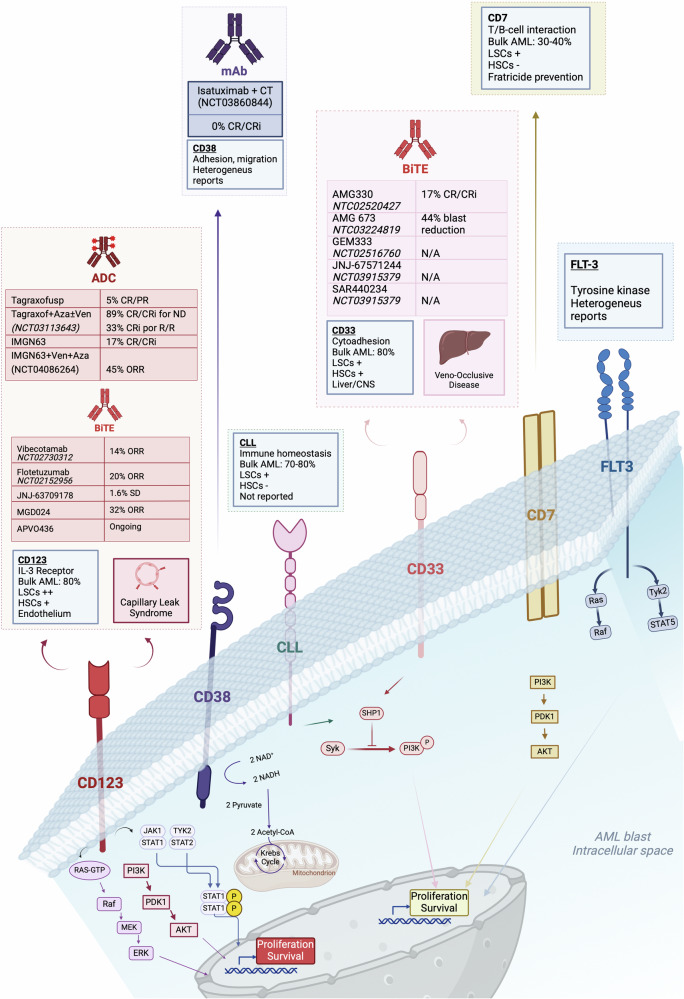
Main characteristics of the most common antigens targeted with current directed therapies for AML and relevant clinical trials. Antibody-drug conjugates (ADC) targeting CD123 shown are Tagraxofusp in monotherapy (208); Tagraxofusp + Azacitidine ± Venetoclax (209); IMGN63 (210); IMGN63 +Venetoclax + Azacitidine (211). Biespecific T-cell Engagers (BiTE) targeting CD123 exposed are Vibecotamab (212); Flotetuzumab (213); JNJ-63709178 (214); and MGD024 (215). Monoclonal Antibodies (mAb) targeting CD38 are isatuximab in combination with chemotherapy (268). BiTE targeting CD33 are AMG330 (233) and AMG673 (235). AZA Azacitidine, Ven Venetoclax, CT chemotherapy, CR complete response, CRi complete response with incomplete hematologic recovery, PR partial response, ND new diagnosed, R/R refractory/relapsed, ORR Overall Response Rate, SD stable disease, This figure was created with Biorender.com

One of the earliest demonstrations of the potential of CAR-T cells for AML was a construct developed by Ritchie and colleagues that targeted the Lewis Y (LeY) antigen.^195^ This CAR-T was tested in a Phase 1 clinical trial, demonstrating a favorable safety profile and durable in vivo persistence. However, the efficacy of this approach was limited, and all patients relapsed with the detection of LeY-positive AML blasts. In current clinical practice, CD33 and CD123 represent the most utilized molecules for CAR-T cell engineering^196^ (Table 2, Fig. 4).

**Table 2:** Current clinical trials with CAR-T cells for AML

Target Antigen	Clinical trial.gov Identifier	Study Summary	Trial Phase	Disease Status	Manufacturer	Status	Location	Reference
CD123	NCT04230265	Multicenter, open-label, adaptive design Phase 1 trial with genetically modified T cells carrying universal CAR (UniCAR02-T) in combination with CD123 target module (TM123) for the treatment of hematologic and lymphatic malignancies positive for CD123	I	R/R AML	GEMoaB Monoclonals GmbH	R	Germany, multicenter	^292,603,604^
CD123	NCT05949125	Multicenter, open-label, Phase 1 study of Allo-RevCAR01-T-CD123 consisting of genetically modified T cells carrying reverse chimeric antigen receptors (Allo RevCAR01 T) in combination with CD123 Target Module (R-TM123) for the treatment of patients with selected hematologic malignancies positive for CD123	I	R/R AML	Cellex GmbH	R	Germany, multicenter	^605,606^
CD123	NCT03190278	AMELI-01. Phase 1, open label dose escalation and dose-expansion study to evaluate the safety, expansion, persistence, and clinical activity of UCART123 (UCART123v1.2) in R/R AML	I	R/R AML	Cellectis	R	US, multicenter	^300^
	NCT04106076	Phase 1, Open Label Dose-escalation Study to evaluate the Safety, expansion, persistence and clinical activity of UCART123 in patients with newly diagnosed CD123 positive adverse genetic risk AML	I	Adverse Genetic Risk AML	Cellectis	WD	N/A	
CD123	NCT05457010	Evaluate the safety and preliminary activity of (Antigen Receptor Complex-T cell platform) ARC-T cells and SPRX002 in R/R AML or HR-MDS	I	R/R AML R/R HR-MDS	AvenCell’s	R	US (MDACC)	
CD123	NCT04318678	CATCHAML: CD123.CD28.ζ-CAR and a CD20 safety switch. CD123-Directed autologous T-cell therapy for AML	I	AML MDS	AvenCell Therapeutics	ANR	US (St. Jude)	^301^
CD123	NCT02159495	Phase 1 Study of T cells lentivirally transduced to express a CD123. CD28.ζ-CAR and a truncated EGFR safety switch. CD123-specific, hinge-optimized, cd28-costimulatory chimeric antigen receptor and a truncated EGFR for patients with CD123 + R/R AML and R/R BPDCN	I	R/R AML BPDCN	Mustang Bio, Inc., in collaboration with City of Hope	ANR	US (City of Hope)	^304,305^
CD123	NCT04272125	Safety and Efficacy of CD123-Targeted CAR-T Therapy for R/R AML	I/II	R/R AML	Chongqing Precision Biotech Co.	R	China (Chongqing University)	-
CD123	NCT04265963	CD123-Targeted CAR-T Cell Therapy for R/R AML	I/II	R/R AML	Chongqing Precision Biotech Co.	R	China (Joint Logistics)	
CD123	NCT03796390	Phase 1 clinical study to evaluate efficacy and safety of CAR-T targeting CD123 in the treatment of AML	I	R/R AML	Hebei Senlang Biotechnology Inc., Ltd.	UN	China (Hebei)	-
CD123	NCT03672851	Study Evaluating Safety and Efficacy of CAR-T Cells Targeting CD123 in Patients With	I	R/R AML/ALL	Nanjing Legend Biotech Co.	T	China (Second Affiliated Hospital of Xi’an Jiaotong University)	-
CD123	NCT03114670	Safety and effectiveness of anti-CD123 CAR-T cells in patients with AML that relapsed after alloHSCT	I	AML reactivated after alloHSCT	Affiliated Hospital to Academy of Military Medical Sciences	UN	China (Affiliated Hospital to Academy of Military Medical Sciences)	-
CD33	NCT03971799	Phase 1/2 study of Anti-CD33 chimeric antigen receptor-expressing T cells (CD33CART) in children and young adults with R/R AML	I/II	R/R AML	Biopharmaceutical Development Program at the Frederick National Laboratory for Cancer Research (NCI)	R	US, multicenter	^306,310^
CD33	NCT03126864	A Phase 1 safety study of adoptive cellular therapy using autologous T cells transduced with lentivirus to express a CD33-specific chimeric antigen receptor in patients with R/R CD33-positive AML	I	R/R AML	MD Anderson Cancer Center	T	US (MDACC)	
CD33	NCT03927261	A Phase 1/1b Safety Study of PRGN-3006 adoptive cellular therapy in patients with CD33-positive R/R AML, Minimal Residual Disease (MRD) positive AML, and HR-MDS	I	R/R AML MDS	Precigen, Inc.	ANR	US (H Lee Moffitt and Mayo Clinic)	^607^
CD33	NCT04835519	Phase 1/2 Study of enhanced CD33 CAR-T cells in subjects with R/R AML	I/II	R/R AML	Beijing Gobroad Boren Hospital	T	China (Beijing Boren Hospital)	^314,608^
CD33	NCT05473221	Evaluate the safety and efficacy of CD33 CAR-T cells in patients with R/R AML	I	R/R AML	Zhejiang University	NYR	China (Zhejiang University)	-
CD33	NCT06326021	Optimized CD33 (FL-33) CAR-T therapy for R/R AML. Phase 1 clinical trial to explore the safety and efficacy of FL-33 CAR T therapy for R/R AML	I	R/R AML	Beijing Gobroad Boren Hospital	R	China (Beijing GoBroad Hospital)	-
CD33	NCT01864902	Treatment R/R CD33 Positive AML by CART-33 (CART33)	I/II	R/R AML	Chinese PLA General Hospital	UN	China (PLA General Hospital)	^609^
CD33	NCT02799680	Treatment of R/R CD33-positive AML by Infusions of allogeneic CART-33	I	R/R AML	Chinese PLA General Hospital	UN	China (Academy of Military Medical Sciences)	-
CD33	NCT05445765	Anti-CD33 CAR-T Cells for the Treatment of R/R CD33 + AML	I	R/R AML	iCar Bio Therapeutics	UN	China (Hebei Yanda Lu Daopei)	-
CD33	NCT05984199	Phase 1/2 study of donor-derived anti-CD33 CAR expressing T cells (VCAR33) in patients with R/R AML after alloHSCT	I/II	R/R AML	Vor Biopharma	R	US (multicentrer)	^610–612^
CD33	NCT05105152	PLAT-08: A Study Of SC-DARIC33 CAR-T cells in pediatric and young adults with relapsed or refractory CD33 + AML	I	R/R AML	2seventy bio	R	US (Seattle Children’s Hospital)	^613^
CD33	NCT05945849	Phase 1 Study of lentivirally transduced T Cells engineered to contain anti-CD33 linked to TCRζ and 4-1BB signaling domains in combination with CD33KO-HSPC in subjects with R/R AML	I	R/R AML	University of Pennsylvania	R	US (University of Pennsylvania)	-
CD33	NCT05942599	Base edited CAR-T cells against AML (BE CAR-33): deep conditioning ahead of alloHSCT (CARAML)	I	R/R AML Bridge to alloHSCT	Great Ormond Street Hospital (UK)	R	United Kingdom (Great Ormond)	-
CD33	NCT04766840	Phase 1study to evaluate the safety and efficacy of CD33 CAR-T in patients with R/R AML	I	R/R AML	Beijing Immunochina Medical Science & Technology Co., Ltd.			
CD123-CD33	NCT04156256	Phase 1, interventional, single arm, open label, treatment study to evaluate the safety and tolerability of CD123-CD33 cCAR in patients with R/R, high-risk hematologic malignancies	I	R/R AML R/R MDS	iCell Gene Therapeutics	UN	China (Peking University)	^614^
CD123 Tim-3	NCT04014881	Application of anti-Tim-3/CD123 CAR-T cell therapy in R/R AML	I	R/R AML	Wuhan Union Hospital	UN	China (Xuzhou Medical University)	-
CD33 or CD123	NCT06420063	Sequential CAR-T cells targeting CD33/CD123 in patients with AML (BAH244)	I/II	R/R AML	Essen Biotech	R	China (Shanghai)	-
CLL1	NCT04884984	Anti-CLL1 CAR T-cell therapy in CLL1 positive for R/R AML	I/II	R/R AML	The First Affiliated Hospital of Soochow University	R	China (Hospital of Soochow)	-
CLL-1	NCT06128044	A Phase 1, multicenter, open-label study of CB-012, a CRISPR-edited allogeneic Anti-CLL-1 CAR-T cell therapy in patients with R/R AML (AMpLify trial)	I	R/R AML	Caribou Biosciences, Inc.	R	US, multicenter	^322,615^
CLL1	NCT05252572	Clinical study of CLL1 CAR-T cells in the treatment of hematological malignancies	I	R/R AML	Zhejiang University	R	China (Zhejiang University)	-
CLL1	NCT05467202	Evaluate the safety and efficacy of CLL1 CAR-T in patients with R/R AML	I	R/R AML	Zhejiang University	NYR	China (Zhejiang University)	-
CLL-1	NCT04219163	Chimeric antigen receptor T-cells for the treatment of AML Expressing CLL-1 Antigen	I	R/R AML	Center for Cell and Gene Therapy, Baylor College of Medicine	R	US (Baylor College)	-
CLL-1	NCT04923919	Clinical study of CAR-T in the treatment of R/R AML	I	R/R AML	920th Hospital of Joint Logistics Support Force of the People’s Liberation Army of China	R	China (Joint Logistics)	-
CLL-1	NCT04789408	A Phase 1 open-label, multicenter study evaluating the safety of KITE-222, an autologous anti-CLL-1 CAR T-cell therapy, in subjects with R/R AML	I	R/R AML	Kite, a Gilead Company	T	US, multicenter	-
CD123/CLL1	NCT03631576	CD123/CLL1. CAR-T cells for R/R AML (STPHI_0001)	II/III	R/R AML	Fujian Medical University	UN	China (Fujian University)	-
CLL1-CD33	NCT03795779	Phase 1, interventional, single arm, open label, treatment study to evaluate the safety and tolerability of CLL1-CD33 cCAR in patients with R/R, high risk hematologic malignancies.	I	R/R AML R/R MDS	iCell Gene Therapeutics	UN	China (Peking University)	^616^
CLL1 + CD33	NCT05467254	Evaluate the safety and efficacy of CLL1 + CD33 CAR-T in patients with R/R AML	I	R/R AML	Zhejiang University	NYR	China (Zhejiang University)	-
CLL1 + CD33	NCT05943314	Clinical study on safety and efficacy of anti-CLL1/ + CD33 CAR T cells in the treatment of AML	I	R/R AML	Guangzhou Bio-gene Technology Co., Ltd	WD	China (Fujian University)	-
Dual CD33-CLL1	NCT05016063	Phase 1 study to evaluate the safety and effectiveness of dual CD33-CLL1 CAR-T therapy in R/R AML	I	R/R AML	Sichuan Kelun-Biotech Biopharmaceutical Co., Ltd.	UN	China (Xinqiao Hospital)	-
CLL1/CD33	NCT05654779	Safety, tolerability, PK, and anti-tumor efficacy of CLL-1/CD33 Targeted LCAR-AMDR cells in subjects with R/R AML	I	R/R AML	Nanjing Legend Biotech Co.	T	China (Institute of Hematology & Blood Diseases Hospital, China)	-
Dual CD38-CLL1	NCT06110208	Phase 1 study to evaluate the safety and effectiveness of dual CD38-CLL1 CAR-T therapy in R/R AML	I	R/R AML	Gracell Biotechnologies (Shanghai) Co., Ltd.	R	China (920th Hospital of Joint Logistics Support Force of People’s Liberation Army of China)	-
FLT3	NCT05023707	Anti-FLT3 CAR-T cell therapy in FLT3-positive R/R AML	I/2	FLT3 + R/R AML	PersonGen BioTherapeutics (Suzhou) Co., Ltd.	R	China (Hospital of Soochow)	-
FLT3	NCT05445011	Safety and efficacy of anti-FLT3 CAR-T cell (TAA05 Cell Injection) in the treatment of R/R AML	I	R/R AML	PersonGen BioTherapeutics (Suzhou) Co., Ltd.	R	China (Wuhan)	^617^
FLT3	NCT03904069	Phase 1 study evaluating the safety, tolerability, and efficacy of FLT3 CAR-T AMG 553 in subjects with FLT3-positive R/R AML	I	R/R AML	Amgen	WD	US (City of Hope, MDACC)	^618,619^
FLT3	NCT05432401	Clinical study of TAA05 injection in the treatment of adult patients with FLT3-positive R/R AML	I	FLT3 + R/R AML	PersonGen BioTherapeutics (Suzhou) Co., Ltd.	R	China (HuaZhong)	-
CD38	NCT04351022	CD38-targeted CAR-T in R/R AML	I/II	R/R AML	The First Affiliated Hospital of Soochow University	UN	China (Soochow)	^323,620^
CD38	NCT05239689	Clinical Study of CD38 CAR-T cells in the treatment of hematological malignancies	I	R/R AML	Zhejiang University	R	China (Zhejiang University)	-
CD38	NCT05442580	Safety and manufacturing feasibility of CART-38 cells in patients with R/R AML or Multiple Myeloma	I	R/R AML MM	University of Pennsylvania	R	US (University of Pennsylvania)	-
CD70	NCT06326463	CAR-T cell therapy directed to CD70 for pediatric patients with hematological malignancies	I	AML, Childhood	St. Jude Children’s Research Hospital	R	US (St. Jude)	-
CD70	NCT04662294	CD70 CAR-T for patients with CD70 positive malignant hematologic diseases	I/II	R/R AML	Zhejiang University	R	China (Zhejiang University)	-
CD19	NCT03896854	Pilot study of the efficacy and safety of CD19 targeted CAR in the treatment of R/R CD19 positive AML	I/II	R/R AML	Shanghai Unicar-Therapy Bio-medicine Technology Co.,Ltd	R	China (Shanghai Bio-medicine Technology)	-
CD19	NCT04257175	Giving CAR-T CD19 transgenic T Cells for AML with t(8;21) and CD19 expression	II/III	R/R AML With t 8:21 and CD19	Sheba Medical Center	R	Israel (Chaim Sheba)	-
CD7	NCT04762485	Humanized CAR-T Cells against CD7 for R/R CD7+ acute leukemia	I/II	R/R AML	PersonGen BioTherapeutics (Suzhou) Co., Ltd.	UN	China (Soochow University)	^260^
CD7	NCT04033302	A multi-center study of multiple CAR-T cell therapy for CD7-positive hematological malignancies	I/II	R/R AML	Shenzhen Geno-Immune Medical Institute	UN	China (Shenzhen Medical Institute)	-
CD7	NCT05995028	Safety and efficacy of universal 4SCAR7U T-cell therapy targeting CD7-positive hematological malignancies	I	R/R AML	Shenzhen Geno-Immune Medical Institute	R	China (Shenzhen Medical Institute)	-
ILT3	NCT04803929	Clinical study of autologous T- cells modified with ILT3 CAR for R/R AML (M4/M5)	I	R/R AML (M4, M5)	Carbiogene Therapeutics Co. Ltd.	R	China (Zhejiang University)	-
ADGRE2	NCT05463640	Evaluate the safety and efficacy of ADGRE2 CAR-T in patients with R/R AML	I	R/R AML	Zhejiang University	NYR	China (Zhejiang University)	-
NKG2D-Ligands	NCT02203825	A Phase 1 study of CAR modified T-cells targeting NKG2D-Ligands (CM-CS1/ CYAD-01) in patients with AML/Advanced MDS-RAEB and MM.	I	R/R AML R/R MDS R/R MM	Celyad Oncology SA	T	US (Dana–Farber)	^327,621^
NKG2D	NCT04658004	NKG2D CAR-T cell therapy for patients with R/R AML	I	R/R AML	Yake Biotechnology Ltd.	NYR	China (Zhejiang University)	-
NKR-2	NCT03612739	EPITHINK: Epigenetic drug treatment and therapeutic immunotherapy with NKR-2	I	AML, Adult	Celyad Oncology SA	WD	No location data Carried by Celyad SA	-
Anti-siglec-6	NCT05488132	Administration of anti-siglec-6 CAR-T cell therapy in R/R AML	I	R/R AML	Xuzhou Medical University	R	China (Xuzhou Medical University)	-
IL1RAP	NCT04169022	Targeting Interleukin 1 Receptor Accessory Protein (IL1RAP) expressing AML cells by CAR-T cells	NA	R/R AML	Centre Hospitalier Universitaire Dijon	T	France (Centre Hospitalier Besançon)	-
MLM-CAR44.1	NCT04097301	A Phase 1-2a trial to assess the safety and antitumor activity of autologous CD44v6 CAR-Tcells in AML and multiple myeloma expressing CD44v6)	I/II	R/R AML MM	IRCCS Ospedale San Raffaele AGC Biologics S.p.A.	T	Italy (IRCCS San Raffaele)	^273,275,622^
IL3	NCT04599543	L3 CAR-T cell therapy for patients with CD123 positive R/R AML	I	R/R AML	Yake Biotechnology Ltd.	NYR	China (Zhejiang University)	-
CD3	NCT05672147	CD33-CAR T cell therapy for R/R AML	I	R/R AML	City of Hope Medical Center	R	US (City of Hope)	-
CI-135	NCT05266950	Safety and efficacy study of CI-135 CAR-T cells for R/R AML	I	R/R AML	Beijing Boren Hospital	R	China (Beijing Boren Hospital)	-
CD4	NCT06197672	CAR-T cell redirected to target CD4 positive R/R AML as a bridge to allogeneic stem cell transplant	I	R/R AML	Brown Center for Immunotherapy	R	US (Indiana University Melvin)	^623^
Tim-3/CD123	NCT06125652	Administration of anti-Tim-3/CD123 CAR-T cell therapy in R/R AML	I/II	R/R AML	Xuzhou Medical University Xuzhou Medical University	R	China (Xuzhou Medical University)	-
CLL-1, CD33, CD38 or CD123	NCT05995041	Universal CAR-T cells targeting CLL-1, CD33, CD38 and CD123 in patients with R/R AML	I	R/R AML	Shenzhen Geno-Immune Medical Institute	R	China (Shenzhen Medical Institute)	-
CD33, CD38, CD56, CD123, CD117, CD133, CD34 or Muc1	NCT03473457	The prospective, multi-center and single-arm clinical study of CAR-T cells therapy in R/R AML	NA	R/R AML	Zhujiang Hospital	T	China (Zhujiang Hospital)	-
Muc1/CLL1/CD33/CD38/CD56 or CD123	NCT03222674	Multi-center Phase 1/2 clinical trial of multi-CAR T cell therapy for AML	I/II	R/R AML	Shenzhen Geno-Immune Medical Institute	UN	China (Guangzhou Medical University)	^624,625^
CLL-1 CD123 or CD33	NCT04010877	Multiple CAR-T cell therapy targeting AML	I/II	R/R AML	Shenzhen Geno-Immune Medical Institute	UN	China (Shenzhen Geno-Immune Medical Institute)	^626^
CD19 BCMA CD123 CD7	NCT05513612	Safety and efficacy of novel autologous CAR-T cells in patients with hematopoietic and lymphoid malignancies.	I	R/R AML	UTC Therapeutics Inc.	WD	China (Shanghai Pudong Hospital)	-

Data from https://clinicaltrials.gov as of September 04 2024. R recruiting, NYR not yet recruiting, ANR active not recruiting, T terminated, WD withdrawn, UN unknown, R/R AML relapse/refractory AML, BPDCN Blastic plasmacytoid dendritic neoplasm, HR-MDS High Risk Myelodysplastic Syndrome

#### CD123

CD123, also known as the alpha chain of the IL-3 receptor (IL-3Ra), is a membrane protein highly expressed in AML (~90% of cases),^197,198^ CD123 has also been identified in other myeloid malignancies, such as myelodysplastic syndrome, chronic myelomonocytic leukemia, chronic myeloid leukemia, and myeloproliferative neoplasms,^199^ CD123 is involved in the JAK, MAPK, and PI3K/AKT signaling pathways, which regulate cell proliferation and differentiation.^200^

Overexpression of CD123 has been identified in up to 95% of LSCs and AML blast cells.^201^ About on-target/off-tumor toxicities, there is controversy over its expression in healthy HSCs. Some reports have identified low CD123 expression patterns on HSCs,^199^ while other preclinical studies have described a myeloablative effect of CD123-directed CAR-T cells.^202^ A significant concern is the potential extra-hematologic toxicities, as CD123 is expressed in endothelial cells, which could result in capillary leak syndrome.^203,204^

Several unconjugated monoclonal antibodies (mAbs), including the CD123-directed mAbs CSL360 and CSL362 (talacotuzumab), have demonstrated limited efficacy in the treatment of R/R AML.^205,206^ Consequently, current strategies predominantly entail the use of toxin-conjugated constructs or Bi-specific T-cell engagers (BiTEs). In this line, various agents targeting CD123 have been developed, including tagraxofusp, a recombinant protein comprising a truncated diphtheria toxin fused to IL-3, which has been approved for the treatment of blastic plasmacytoid dendritic cell neoplasm (BPDCN).^204,207^ Tagraxofusp has demonstrated preliminary clinical activity in the context of AML.^208^ Additional strategies targeting CD123, including BiTEs such as pivekimab sunirine (PVK, IMGN632),^209,210^ vibecotamab^211^ and flotetuzumab^212,213^; as well as other strategies such as JNJ-63709178, MGD024,^214^ and APVO436,^215^ are currently under investigation, with promising results *(*Fig. 4*)*.

#### CD33

CD33 is a transmembrane receptor stimulated by sialic acid residues that is expressed in approximately 85% of AML cases.^216^ CD33 undergoes internalization and dimerization upon binding, making it an attractive therapy and drug delivery target. Its primary role is related to the downregulation of cytokine production and monocyte activation.^217,218^ It is known to be present in over 85% of LSCs, with relatively low expression in HSCs.^216^

The humanized anti-CD33 antibody drug-conjugated gemtuzumab ozogamicin is EMA and FDA-approved for first line use in combination with intensive chemotherapy in treatment-naïve AML.^219–224^ The primary toxicities reported are persistent thrombocytopenia and veno-occlusive disease (VOD).^225^ The latter was initially associated with CD33 expression in Kupffer cells within hepatic sinusoids following FDA approval in 2000.^226^ Upon reapproval in 2017 for administration in a fractionated dosing schedule, VOD was less frequent and was subsequently reported to be associated with the direct hepatotoxicity of the conjugated drug (calicheamicin).^224^

In line with the evidence reported with anti-CD123 unconjugated molecules, mAbs and antibody-drug conjugates directed against CD33 (lintuzumab,^227–229^ SGN-CD33A,^230^ and IMGN779^231^) have failed to demonstrate relevant antileukemic activity to date. Notably, several CD33 × CD3 BiTEs have shown promising results with an acceptable toxicity profile in clinical trials for R/R AML (AMG 330,^232,233^ AMG 673,^234^ GEM333, JNJ-67571244, and SAR440234) (Fig. 4).

#### CLL-1

CLL-1, also known as CLEC12A, myeloid inhibitory c-type lectin-like receptor,^235^ dendritic cell-associated C-type lectin 2 (DCAL-2),^236^ or CD371, is a C-type lectin-like type II transmembrane receptor with a role in cell adhesion and cell-to-cell signaling.^237–239^ It is expressed in over 85% of patients with AML, both in blasts and LSCs.^235,240^ Furthermore, it is expressed exclusively in myeloid lineage cells^198^ and is absent in granulocyte-macrophage progenitors. In addition, CLL-1 does not show relevant expression on HSCs or extra-hematological tissues,^239,241,242^ limiting potential on-target/off-tumor risks and making it an exciting target for investigation.^198^

#### NKG2D

Natural killer group 2 member D (NKG2D) is a highly conserved activating receptor of NK cells and T lymphocytes that plays a pivotal role in immune recognition of tumoral cells through the engagement with a group of ligands, namely MICA, MICB, and the UL16-binding proteins.^243–245^ NKG2D ligands are induced in response to DNA damage, inflammation, and malignant transformation.^246–249^ Expression of NKG2D ligands has been documented in different hematologic malignancies, including AML, whereas expression is generally absent in healthy tissues.^250,251^ Consequently, there is growing interest in NKG2D and NKG2D ligands as potential targets for CAR-T cell therapy.^148^

#### CD7

CD7 is a transmembrane protein essential for T cell and T cell/B cell interactions in early lymphoid development.^252,253^ It is among the earliest T cell-associated antigens expressed during T-lymphocyte maturation and is aberrantly present in approximately 30-40% of adult AML patients.^254,255^ An important consideration is that CD7, present on T cells, requires CD7-directed CAR-T cells with CD7 gene-knockout strategies to prevent effector T cell fratricide.^256–259^ Some strategies have been successful in preliminary studies, mainly for T cell leukemia/lymphoma.^255,260^ Consequently, CD7 may represent a viable target for CD7 + AML.^253,258,259^

#### CD38

CD38 is a surface glycoprotein that plays a role in cell adhesion, migration, and intracellular calcium mobilization.^261–263^ It is known to be expressed in plasma cells but is also expressed heterogeneously in myeloid cells.^264^ Combinations of the humanized mAb targeting CD38, daratumumab, with other drugs, have demonstrated efficacy in the treatment of AML and T-ALL in clinical trials (NCT03067571 and NCT03384654), with no significant cytopenia reported.^265^ Similarly, another CD38 mAb, isatuximab,^266,267^ has been employed in a second-stage study (NCT03860844), investigating its use in combination with standard salvage chemotherapy in children with R/R ALL or AML in first or second relapse. No unexpected safety issues were observed, and complete response (or complete response with incomplete peripheral blood count recovery) was observed in 50% of patients in the AML cohort.^268^ Then, targeting CD38 presents a promising strategy that may additionally hold potential benefits for a variety of malignancies.^269^

#### CD44v6

CD44, or the receptor for hyaluronic acid, is a cell surface adhesive molecule implicated in the aggregation, migration, activation, and malignant transformation of leukocytes.^270–272^ It is expressed on multiple tissue types, although some splice isoforms of CD44 are relatively tumor-restricted. For example, the splice site variant CD44v6 is present in over 60% of AML samples and is not shared by non-tumoral cells^273^ or HSCs, indicating that this antigen is a promising target. The available data demonstrate promising results in in vitro and in vivo mouse models.^273–275^

#### CD70

CD70, or the tumor necrosis factor receptor ligand, is expressed on antigen-presenting cells and is upregulated to promote an effector CD8 + T cell response and downregulate Th17 function. Reports have identified CD70 expression in 45% of AML blasts and in 30% of LSCs,^276^ or even lower.^277^ Other studies suggest its expression in up to 75% of AML LSCs.^199^ The targeting of CD70 with a mAb (cusatuzumab) has been demonstrated to successfully eliminate AML-LSCs when combined with HMAs, making it an exciting target for CAR-T cell therapy.^278^ The preclinical evidence for CAR-T cells targeting CD70 is encouraging.^277,279^

#### FLT3

FLT3 is a very well-characterized receptor ligand for HSCs maintenance and differentiation. It is implicated in 30% of patients with AML (approximately 24% involving FLT3-ITD and 7-10% involving FLT3-TKD^280^). Some FLT3 inhibitors have already received FDA approval (midostaurin, gilteritinib) with an acceptable safety profile.^281^ Additional FLT3 inhibitors under investigation include quizartinib,^282^ sorafenib^283^ and crenolanib.^284^ To date, most data regarding CAR-T cell therapy is preclinical,^285–287^ and significant concerns have been raised regarding FLT3 expression in HSCs. In this context, some constructs in development include safety switches (described in the following sections) to reduce myeloablation.^288^

Other potential targets currently under investigation include CD19,^289^ ADGRE2,^193,290^ and ILT3.^291^ However, there is limited data available regarding their efficacy and safety.

## Current strategies implemented in clinical trials

Currently, there are 75 registered clinical trials of CAR-T cell therapy for AML (Table 2), of which 53 (71%) are Phase 1 or early Phase 1 trials, with CD33 and CD123 being the most frequently targeted antigens (Fig. 4). Most of these trials recruit patients from China and the US. Past and ongoing clinical trials of CAR-T cells for treating AML are discussed below.

### CD123-CAR-T cells in clinical trials

In 2020, a German group testing CAR-T cell therapy directed to CD123 reported the preclinical efficacy of UniCAR-T-CD123,^292^ a two-component, rapidly switchable and second-generation CAR-T. The construct carries a CD28 co-stimulatory domain that, by itself, does not recognize any surface antigen apart from a soluble adaptor named targeting module (TM), which is included in the second component.^293,294^ The TM is administered intravenously and confers specificity against the antigen of choice. In this case, the TM included a scFv directed to the CD123 antigen (Fig. 3). Due to the brief half-life of the TM, the interruption of its continuous intravenous administration rapidly deactivates T cell activity, which mitigates the associated toxicities.

This strategy is currently being investigated in a Phase 1a trial in R/R AML (NCT04230265), with early promising results and an acceptable toxicity profile. Indeed, the trial was modified to include a prolonged schedule of TM administration. To date, 19 patients who have undergone extensive prior treatment have received UniCAR-T-CD123, including 12 with previous alloHSCT. CRS was observed in 12 patients, mainly grade 1 or 2. No prolonged myelosuppression was observed, and therefore rescue alloHSCT was not required. The ORR for the R/R AML population was 53%.^295^ The same group developed the world’s first CRISPR-engineered switchable allogeneic CAR-T designed to circumvent graft-versus-host disease (GvHD) and cell rejection. The trial is currently in progress and is registered as Phase 1 (NCT05949125).^296^

A multicentric clinical trial (NCT03190278) conducted in the US has evaluated the efficacy of universal CD123-directed CAR-T cells. This approach involved gene-editing technology to modify allogeneic T cells to express a second-generation CAR targeting CD123 (comprising CD123 scFv-4-1BB-CD3ζ).^297^ The TCR αβ was inactivated by targeting the TRAC gene using TALEN® nucleases, which helps to reduce the risk of GvHD. Furthermore, a “safety switch” was incorporated by including an RQR8 deletion ligand containing epitopes from CD34 and CD20 antigens, thereby conferring susceptibility to rituximab on the modified cells.

The CAR-T cell product demonstrated potent antitumor activity in vitro and long-term disease-free survival in a subset of primary patient-derived BPDCN xenograft (PDX) mouse models.^298^ Concerns arose during the transition to the clinic when the first patient treated (UCART123; NCT03203369) died after developing severe CRS and pulmonary capillary leak syndrome.^299^ The trial was discontinued and later reopened for R/R AML, with the addition of a CD52 knockout, dose reduction, and an upper age limit (AMELI-01; a Phase 1 open-label dose-escalation trial testing UCART123v1.2), which is still recruiting (NCT03190278, NCT04106076). Preliminary data presented at ASH (American Society of Hematology) 2022 congress indicated that the addition of alemtuzumab to the fludarabine and cyclophosphamide lymphodepletion regimen resulted in more robust and greater UCART123v1.2 expansion, which was an essential concern in an allogeneic context. Moreover, the expansion of UCART123v1.2 correlated with a reduction in tumor burden.^300^ CRS occurred in 100% of the cohort, with some cases being severe, and therefore, systematic tocilizumab is planned to be administered for future stages.

Alternative approaches consider CAR-T cell treatment in the context of a bridge to alloHSCT. One example is the CATCHAML trial (NCT04318678) for pediatric R/R AML. This involves a second-generation CD28 co-stimulated CAR that incorporates a CD20 safety switch (Fig. 3). To date, preliminary data from 12 patients enrolled in the trial report the absence of grade ≥2 CRS or neurotoxicity despite the heterogeneous nature of the responses observed. A phenotypic analysis conducted by the researchers revealed that CD123-CAR-T cell products were predominantly effector memory cells. Accordingly, the next generation of CD123-CAR T-cell products will be manufactured in the presence of dasatinib, with the objective of limiting T cell differentiation and exhaustion.^301^

Several ongoing trials are employing additional approaches targeting CD123. There is compelling preclinical evidence from a soluble tumor-targeting protein (SPRX002) that exclusively binds to AML cells expressing CD123, thereby enabling their destruction by T cells transduced ex vivo. These are known as Antigen Receptor Complex (ARC) T cells.^302^ A Phase 1 clinical trial (NCT05457010) is currently enrolling and is projected to conclude in November 2025. A strategy involving the electroporation of anti-CD123-CAR mRNA into “biodegradable” T cells in patients with R/R AML (NCT02623582) was terminated due to an absence of an anti-tumor effect.^303^ Finally, a case report from a pivotal trial testing a fourth-generation, apoptosis-inducible lentiviral CAR targeting CD123 has been published,^262^ but there is no further evidence of this construct.

More conventional strategies such as CD123CAR (autologous CD123CAR-CD28-CD3ζ-EGFRt) have demonstrated antileukemic efficacy with acceptable toxicities in seven patients with R/R AML who had undergone prior alloHSCT (NCT02159495).^304,305^

### CD33-CAR-T cells in clinical trials

A second-generation CD33-directed CAR construct comprising lintuzumab-CD28/CD3ζ^306^ has demonstrated promising outcomes in a Phase 1/2 dose-escalation interim analysis of children, adolescents, and young adults with R/R AML (NCT03971799). This construct includes a combination of a targeting motif derived from lintuzumab (HuM195, SGN-33; an antibody that targets the distal CD33 IgV domain) linked to CD28/CD3. The rationale was based on the observation that the rs12459419 C > T single nucleotide polymorphism, associated with decreased CD33 surface expression, is present in more than 50% of AML patients.^307–309^ The lintuzumab-enhanced CD33 CAR-T cells are capable of recognizing low-antigen-density AML.

A total of 24 subjects were enrolled in the trial, of whom 12 had undergone prior alloHSCT. CD33-CAR-T products were successfully manufactured for 23 patients and subsequently infused into 19, primarily due to the progression of the disease. Four patients experienced CRS grade 3 or 4, which was successfully managed. Responses were reported in those who achieved the highest dose level. Based on the rapid clinical efficacy observed at dose level 4, enrollment has resumed in Phase 2.^310^ The authors highlight that despite the considerable inter-patient heterogeneity of apheresis products, the centralized manufacturing of CD33-CAR-T cells was feasible for the 6 participant centers.^311^ Notably, the authors did not report sinusoidal obstruction syndrome, a complication that has been previously associated with gemtuzumab.

A Phase 1 clinical trial conducted at MD Anderson Cancer Center evaluated the efficacy of a 4-1BB and CD3ζ endodomain co-expressed with a truncated human epidermal growth factor receptor but failed to demonstrate an anti-leukemic effect (NCT03126864). The researchers encountered difficulties regarding the efficiency of transduction and the complexity of the clinical setting, given the 2–4 weeks required for production (risk of AML progression and other clinical complications such as infections). As a consequence of this, the trial was closed after the enrollment of 11 patients.^312^ The current focus of research is on the development of a platform that will facilitate more rapid production and in vivo expansion of a product referred to as PRGN-3006 or UltraCAR-T. The preliminary responses were encouraging,^313^ reason why the product gained the fast-track designation by the FDA. The UltraCAR-T is based on a non-viral Sleeping Beauty system to express the CAR, as well as a membrane-bound IL-15 for stronger in vivo expansion and persistence. Furthermore, the construct contains a safety switch that can conditionally eliminate CAR-T cells, thereby improving the toxicity profile (Fig. 3).

The addition of a potentiating molecule linked to human CD33 scFv *via* a self-cleaving P2A peptide has been reported to functionally enhance CD33-CAR-T cells and render them safe. Furthermore, this approach has demonstrated anti-leukemic efficacy, as revealed in a preliminary report at ASCO 2024^314^ (NCT04835519). An intriguing strategy comprising a combination of VCAR33 (allogenic CARs with a lintuzumab-derived binding domain and a CD28 co-stimulatory domain) is currently enrolling patients in a Phase 1/2 study^315^ (NCT04849910), with the possibility of subsequent CD33-deleted alloHSCT consolidation.

An additional conceptually appealing approach may be the drug-induced dimerization of split CAR designs, which allow for cycles of tumor killing interspersed with periods of myeloid recovery. These strategies may permit physicians to modulate CAR activity based on clinical needs. In this context, a CD33-targeted dimerizing agent-regulated immunoreceptor complex (DARIC) is currently being evaluated.^275^ The platform comprises separate antigen targeting and T cell signaling components, with embedded extracellular rapamycin-dependent heterodimerizing domains (Fig. 3). The targeting and signaling components undergo dimerization in the presence of rapamycin, thereby eliciting antigen-responsive T cell activation. Appelbaum et al. reported evidence of in vitro and in vivo antitumor activity against established CD33+ human tumor xenografts in NSG (NOD scid gamma) mouse models.^316^ However, the Phase 1, PLAT-08 trial of SC-DARIC33 in pediatric patients with AML was put on hold by the FDA following a severe grade 5 adverse event report.

Finally, an interesting study performed multiplexed base editing to remove TCR, CD52, and the shared AML/T lineage antigen CD7 from T cells, which enabled the generation of universal donor CAR-T cells (BE-CAR33, BE-CAR7) for combinational use. Preclinical data demonstrated the robust activity of BE-CAR33 alone and in combination with BE-CAR7 against human CD7 + CD33 + AML cells in a PDX model from a KIT-mutated AML patient.^317^ Preliminary results from other early clinical trials of CAR-T cell strategies targeting CD33 have shown promising results with acceptable safety profiles.^141,313,318^

### CLL1-CAR-T cells in clinical trials

A second-generation murine anti-CLL-1 scFv with an intracellular domain of CD28 and OX40 and a PD-1 silencing shRNA sequence, to strengthen CAR-T function, has been tested in a Phase 1 trial. Two patients who had previously undergone anti-CD38 CAR-T cell therapy and experienced treatment failure achieved a complete response, although no further follow-up has been provided.^319^ In a similar strategy, the CB-012 platform has been engineered with next-generation Cas12a CRISPR hybrid RNA-DNA (chRDNA) genome-editing technology to target both checkpoint function and immunomodulation, which has the potential to improve antitumor activity (AMpLify Phase 1 clinical trial^320^) (Fig. 3). In other preliminary studies, CAR-T cells directed against the CLL1 antigen have also been reported to be safe and provide a promising response rate in heavily pretreated patients^321,322^

### Other targets in clinical trials

CD38 has been targeted in a Phase 1/2 CD38-CAR-T cell therapy in six patients with R/R AML post-alloHSCT (NCT04351022). CD38-CAR-T cells were manufactured without incident, with four products derived from an autologous source and two from a donor source. Four weeks after the infusion of CD38-CAR-T cells, the ORR was 66.7% (4/6 patients) including one patient who achieved complete remission (CR) and three patients who achieved complete remission with incomplete count recovery (CRi) and full donor chimerism. The 6-month OS and leukemia-free survival rates were both 50%, with a median of 7.9 and 6.4 months, respectively. Toxicities were considered acceptable.^323^

Some interesting strategies are being explored that target CD7. A recent study involving seven patients with R/R AML suggested that sequential treatment with CD7-CAR-T cells followed by haploidentical HSCT may be both safe and effective, leading to durable responses.^324^ Other strategies include autologous nanobody-derived fratricide-resistant CD7-CAR-T cells. Notably, an off-target CD7 blockade strategy was developed employing a tandem CD7 nanobody VHH6 coupled with an endoplasmic reticulum/Golgi-retention motif peptide, effectively sequestering the CD7 surface marker intracellularly^325^ *(*Fig. 3*)*.

A Phase 1 dose-escalation study was conducted to evaluate the efficacy of NKG2D-CAR-T cells for R/R AML and high-risk myelodysplastic syndrome without lymphodepletion conditioning (NCT02203825). However, no notable expansion or objective clinical efficacy was observed. The possible explanations for the observed results include the lack of a costimulatory domain in the CAR construct, heterogeneous NKG2D ligand expression in the patients, and the conserved nature of NKG2D, which may be related to the lack of immunogenicity.^326,327^

Finally, a Phase 1 trial is currently evaluating a bispecific CLL1-CD33-CAR with two complete CAR constructs connected by a P2A cleavable linker (NCT03795779). Nine patients were treated until September 2019; eight treatments were manufactured from autologous cells, while a ninth was derived from an HLA-matched sibling donor. Two patients experienced grade 3 or 4 CRS, four patients experienced immune effector cell-associated neurotoxicity syndrome (in 3 cases, grade 3 or more), and all patients experienced grade four pancytopenia. On disease re-evaluation at four weeks post-CAR-T cell infusion, seven out of nine patients were negative for minimal residual disease by flow cytometry, and two patients had no response.^328^

## Future directions

### Manufacturing

The primary elements of the CAR-T cell manufacturing process have largely been standardized. They can be divided into four main stages: isolation and enrichment of T cells, activation and expansion of T cells, gene transfer of a CAR vector, and ex vivo CAR-T cell expansion and cryopreservation.^329^

In the first stage, PBMCs (Peripheral Blood Mononuclear Cells) are isolated from peripheral blood by density gradient centrifugation to remove granulocytes, red blood cells, and platelets. In this initial phase, the patient with R/R AML may experience prolonged cytopenia, which could impede the success of apheresis. Notably, the cellular composition at the outset of production can impact the phenotype of the CAR-T cells. At this stage, it should be considered that patients with R/R AML may present with low numbers of effector T cells, besides an activated circulating cytokine profile, as reported in other hematological cancers at advanced stages.^330^

**Fig. 5:**
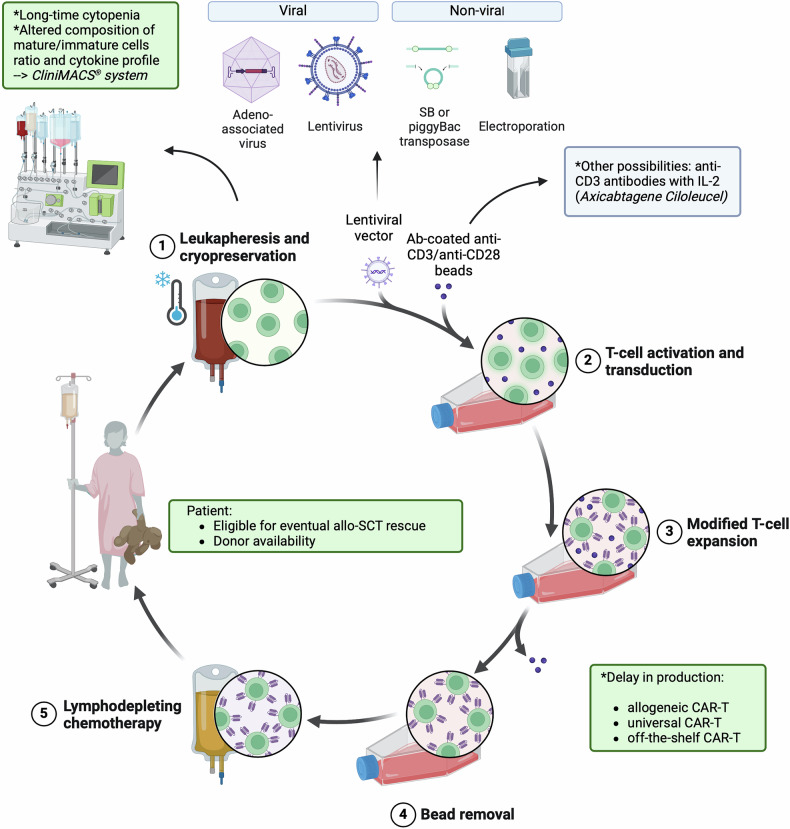
Automated CAR-T cell manufacturing process including leukapheresis and cryopreservation (1), T-cell activation and transduction (2), modified T-cell expansion (3), and lymphodepleting chemotherapy prior to patient infusion. Potential challenges in the AML context are highlighted in the green boxes. This figure was created with Biorender.com

An alternative approach in this context is the selection of CD3 + T cells using magnetic bead-based systems, such as the CliniMACS® system. This enables T cell expansion and administration of the final cell product, ensuring a properly defined CD4:CD8 ratio^331–333^ (Fig. 5).

T cell activation is typically initiated by unconjugated mAbs, most commonly anti-CD3/anti-CD28 antibody-coated magnetic beads.^334^ Afterward, the method of delivery of the CAR transgene can significantly impact its expression level. At present, all FDA- and EMA-approved CAR-T cell products employ lentiviral or retroviral transduction to achieve CAR transgene integration. Still, several alternative non-viral gene delivery methods have been investigated, with promising results.^335^

It is important to note that production delays may prove fatal for this highly aggressive disease. Using donor-derived CAR-T cells, also known as allogeneic CAR-T, universal CAR-T (UCART), or off-the-shelf CAR-T,^336,337^ could expand this strategy by being immediately available. Some of the currently engineered CAR-T cells, such as BE-CAR33 or BE-CAR7, are already based on this strategy and have shown efficacy in PDX models and are currently being tested in the CARAML clinical trial (NCT05942599).^317^

Furthermore, the optimal lymphodepleting therapy remains to be determined. Some evidence supports the use of HMAs concomitantly with the lymphodepleting regimen, which have been demonstrated to augment CD123 and other antigen expression on AML blast surfaces. Recent findings also suggest that combining CAR-T cells with cytokine signaling inhibitors could enhance immunotherapy efficacy in the specific AML context (NCT03766126).^160^

Finally, selecting eligible AML patients may require a more cautious approach than that employed in CAR-T cell therapy for other hematological malignancies. In the event of myeloid aplasia, the patient may require a rescue alloHSCT. Accordingly, a meticulous and multidisciplinary clinical strategy, including the identification of HSCT donors prior to CAR-T infusion, may be considered.

### Strategies to circumvent antigen heterogeneity

Given the molecular and cytogenetic heterogeneity of AML, a promising approach for effective CAR-T cell therapy with increased specificity might be to target two different antigens using tandem or bicistronic CARs. These strategies are still in the early stages of clinical development, with limited available information. Related to this, the emergence of antigen-low expression clones evading effector cell-mediated killing may constitute one mechanism of disease escape during AML evolution.^196^ In this context, both modifying the binding affinity and avidity of the scFv, and selecting appropriate costimulatory domains, can be used to enhance CAR sensitivity.

The scFv determines the antigen-binding affinity and specificity of CAR-T cells.^338^ A high affinity scFv enhances the binding strength to the tumor antigen, improving tumor recognition and cytotoxicity. Additionally, the multivalent nature of CAR constructs facilitates a high-avidity effect, increasing overall binding efficiency and T cell activation, even in the presence of antigen heterogeneity. Optimizing both affinity and avidity can lead to more effective and sensitive CAR-T therapies, enabling better tumor targeting and improved clinical outcomes. However, achieving the right balance of affinity and specificity is complex. The affinity must be strong enough for the CAR-T cells to recognize tumor antigens and induce T cell-mediated destruction, but excessively high affinity may lead to off-target binding to healthy tissues.^339,340^ Furthermore, high-affinity CAR-T cells may exhibit reduced persistence in vivo due to activation-induced T cell exhaustion and apoptosis.^341^

Costimulatory elements may also influence CAR sensitivity. CD28 CARs typically exhibit a reduced requirement for target antigen density,^342^ as well as enhanced and accelerated expansion.^343^ These features make CD28 an interesting co-stimulator domain for the treatment of AML. Indeed, most strategies currently being tested in early clinical stages (discussed in this review) have selected the CD28 co-stimulator domain. Conversely, 4-1BB CARs typically exhibit more remarkable persistence, in part due to the reduction in T cell exhaustion induced by prolonged CAR signaling.^344^ This attribute may be particularly advantageous in contexts where an alloHSCT rescue is not anticipated as a component of the therapeutic strategy.

Additionally, the signaling machinery can be enhanced by modifying the multimeric CD3 complex (CD3 complex-based receptors). This CD3 complex plays a pivotal role in TCR-mediated antigen recognition.^345,346^ Regarding this, preclinical evidence indicates that the substitution of chimeric V_L_–Cα and V_H_–Cβ chains for the endogenous TCR may improve sensitivity.^347^ This is the case of HIT receptors, STAR receptors,^348,349^ TCAR, antibody-TCR (AbTCR), TCR fusion constructs (TRuC),^350^ and T cell antigen coupler (TAC) receptors. Specifically, STAR-T cells have demonstrated superior killing in low neoantigen density context and improved tumor control in mouse models in comparison with conventional CAR-T strategies.^351,352^ However, in contrast to CAR-T cells, most TCR-engineered T cells can only recognize intracellular tumor antigens presented by MHC molecules.^353,354^ Of note, CAR antigen sensitivity may also be enhanced without a structural modification by amplifying downstream activation signaling.^355^

**Fig. 6:**
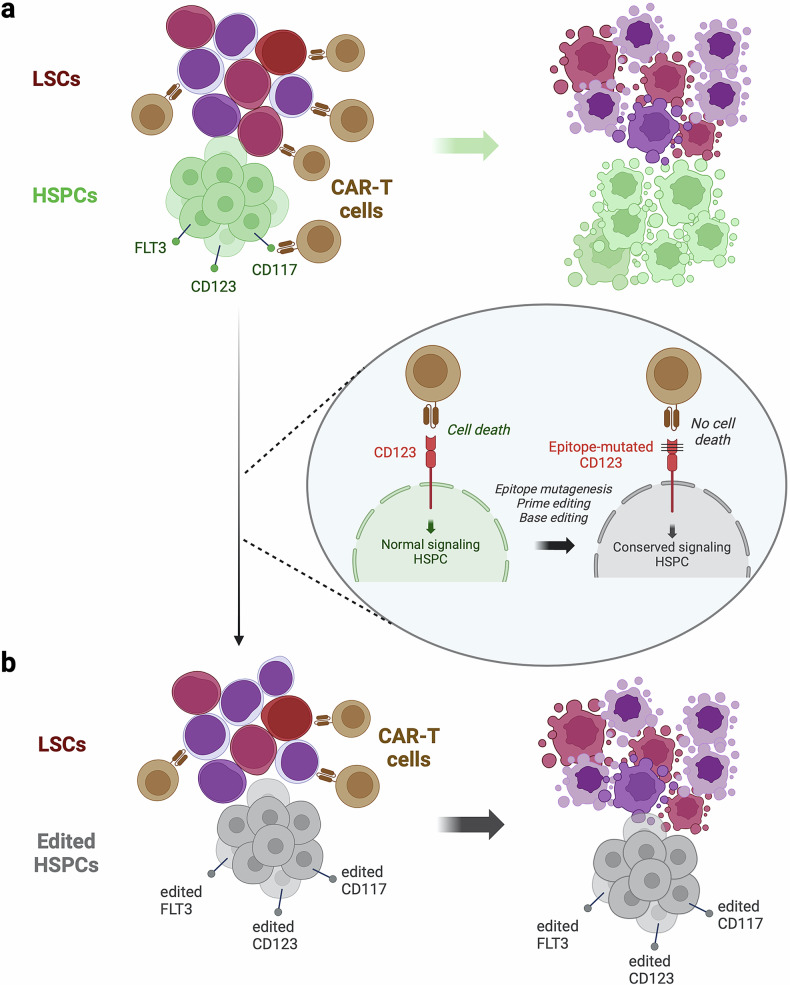
Epitope engineering of Hematopoietic Stem Progenitor Cells (HSPCs) (**a**) to confer resistance to CAR-T cells targeting (**b**). The complexity of AML treatment may require innovative strategies, including recently implemented approaches such as epitope editing through epitope mutagenesis, prime editing, and base editing. LSCs, Leukemia Stem Cells. Figure created with Biorender.com

The complexity of AML treatment may necessitate innovative strategies such as epitope-editing (Fig. 6). Early efforts in epitope-editing included CRISPR/Cas9-mediated CD33 knock-out in CD33-positive tumor cells and primary HSPCs, demonstrating its feasiblility.^356–358^ More recent advancements, such as the work led by Casirati et al., successfully performed epitope engineering of donor CD34+ HSPCs to confer resistance to CAR-T cells targeting FLT3, CD123, and KIT antigens.^359^ The authors confirmed the resistance of epitope-edited hematopoiesis and the subsequent eradication of PDXs after CAR-T cell treatment. In the case of CD123, these results have been validated by other groups.^360^ Similarly, Wellhausen et al. generated CRISPR epitope-edited CD45-CAR-T cells to evade CAR-T cell recognition. These epitope-edited CD45-CAR-T cells exhibited fratricide resistance and efficacy against PDX AML.^361^ Thus, ex vivo epitope editing in HSCs and T cells could revolutionize CAR-T cell therapies for AML, offering a promising strategy to enhance treatment effectiveness and durability.

**Fig. 7:**
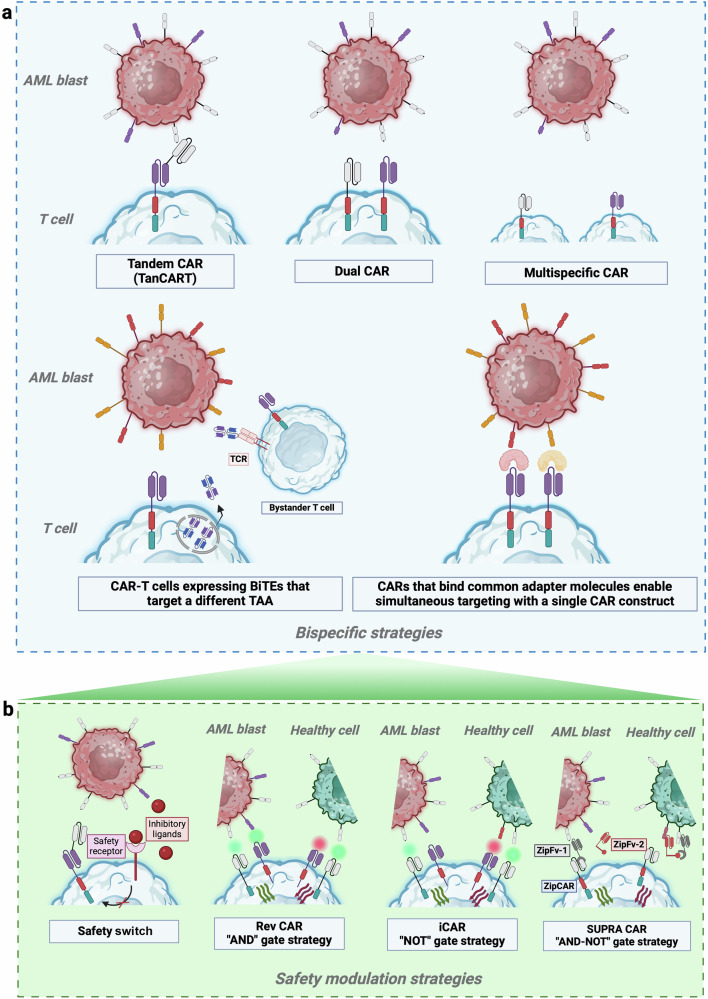
Bispecific strategies (**a**) and safety modulation approaches (**b**) currently under investigation. Ongoing developments in bispecific strategies include tandem CARs, dual CARs, multispecific CARs, and CAR-T cells engineered to express BiTEs targeting a different antigen through the activation of bystander T cells. Another approach involves CARs that recognize a common adapter molecule, allowing for simultaneous targeting with a single CAR construct. In the AML context, key safety strategies include the implementation of safety switches and the use of gating mechanisms, such as “AND,” “NOT,” and “AND-NOT” gate strategies. Figure created with Biorender.com

When considering bispecific and complex editing approaches, it is crucial to control over cell killing to mitigate potential on-target/off-tumor toxicities, particularly in the context of AML (Fig. 7). One of the earliest strategies proposed to regulate toxicity is the use of switchable CAR-T cells. This approach involves introducing genes encoding surface proteins, antigens or intracellular effectors into the CAR-T cells. Upon gene expression, CAR-T cells become responsive to specific drugs, enabling the controlled cessation of their activity.^362,363^ An illustrative example is incorporating a truncated epidermal growth factor receptor (EGFRt) into CAR-T cells. The administration of cetuximab can target EGFRt, which could lead to the elimination of active CAR-T cells through antibody-dependent cellular cytotoxicity.^364^

A novel strategy recently published by He et al.^224^ involves the isolation of multiple nanobodies (heavy-chain-only antibodies with a small single variable domain) that bind to various epitopes. By employing a sequentially tumor-selected antibody and antigen retrieval (STAR) system, the researchers developed a bispecific and split CAR (BissCAR) targeting CD13 and TIM3. The BissCAR-T cells effectively eradicated patient-derived AML in murine and PDX models with limited toxicity to normal HSPCs.

Other relevant platforms currently in development include a split, universal, and programmable (SUPRA) CAR system, which is designed to improve specificity and controllability,^365^ and the RevCAR-T platform, which employs an “AND” logic gate to target CD33 and CD123 in a versatile manner.^366^ In this context, the incorporation of the “OR” or “AND” logic gate strategies could also prove beneficial in reducing off-tumor side effects, as the tumor with both antigens will be selectively eliminated compared with tumors with a single antigen.^367–370^

The “OR” logic gate is based on two completely independent CAR molecules able to recognize the presence of a single antigen or both antigens simultaneously.^182^ The SUPRA CAR system employs an AND or NOT logic gate design to target CD33 and FLT3. The construct comprises of a universal chimeric receptor expressed on the T cell, zipCAR, coupled to a soluble antigen-binding adapter, zipFv, which contains a leucine zipper and scFv.^370^ The “AND” logic gate would only achieve full activation status upon binding their cognate antigens simultaneously. Two distinct CARs are co-expressed on a single T cell, each with complementary signaling domains. However, the CAR-T cell attains full activation only when both of its receptors bind to their respective antigens simultaneously. For example, the use of synthetic Notch (synNotch) receptors in a dual CAR-T system enables synergistic and selective binding, minimizing on-target/off-tumor toxicity.^371–374^ Upon recognition of the antigen by the first receptor, a transcription factor is activated, which in turn induces the expression of a second CAR. This subsequently binds to the secondary antigen.^338^

In addition to these two concepts, “NOT” gates are also noteworthy for their ability to turn off CAR-T cell activity upon encountering unintended target cells. For example, the “NOT”-gate CD93-CAR-T is an inhibitory CAR strategy designed to mitigate endothelial toxicity generated by CD93-CAR-T cells, which eliminates AML but exerts on-target/off-tumor toxicity to endothelial cells.^375^ “IF-THEN” gates (which allow spatiotemporal regulation of CAR expression) and “IF-BETTER” gates (in which a CAR-engaging antigen A performs better in the presence of antigen B) are also under investigation.^347^

Using different logic gate CAR-T designs could also mitigate AML relapse.^376^ Traditional CAR-T therapies can be limited by the heterogeneous nature of AML, where tumor cells may downregulate or lose expression of the targeted antigen, leading to relapse. By employing logic gate circuits, these therapies can be adapted to recognize more complex antigen expression patterns specific to AML.^377,378^ For example, incorporating “OR”-gate CARs – where two independent antigen recognition domains enable targeting multiple antigens – can enhance tumor recognition and reduce the likelihood of escape. Additionally, integrating a “safety switch” that responds to both tumor-associated markers and inhibitory signals from the TME can improve CAR-T cell persistence while minimizing off-target toxicity issue.^56^ Furthermore, optimizing costimulatory domains is essential to ensure adequate CAR-T cell proliferation and persistence, which are strongly correlated with durable leukemia remission.^379^ This multi-layered approach holds significant potential for reducing relapse rates and improving long-term outcomes in AML.

### New antigens under development

**Fig. 8:**
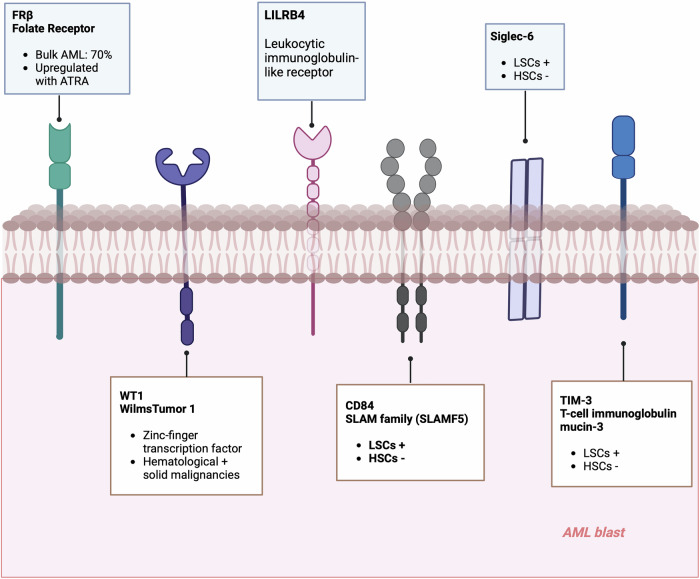
Folate Receptor (FR), Wilm Tumor 1 (WT1), Leukocytic immunoglobulin-like receptor (LILRB4), CD84, Siglec-6, and T-cell immunoglobulin mucin-3 (TIM-3) are antigens that show promising features for CAR-T cell targeting. Figure created with Biorender.com

The major challenge encountered in CAR-T cell development for AML has been the inability to identify a specific targetable antigen. Further efforts are needed in whole-genome sequencing, surfaceome profiling of AML LSCs, and proteomic and transcriptomic studies comparing antigen expression in LSCs and healthy stem cells.^17,146,380^ Some antigens that have been the subject of early directed and promising strategies are shown in Fig. 8.

In a notable advance, Lynn et al.^381^ developed an FRβ-specific CAR construct, supported by compelling preclinical evidence, targeted to a folate receptor (FR). The FR is expressed on approximately 70% of primary AML blasts.^382^ FRα and FRβ are cell surface-bound proteins through glycosyl-phosphatidylinositol linkages. This receptor is an attractive target because its expression is limited in normal tissue and can be upregulated by all-trans retinoic acid,^383^ a drug approved for acute promyelocytic leukemia.

Additionally, Rafiq et al.^384^ successfully developed and tested WT1-CAR-T cells, demonstrating efficacy against cell lines in an in vitro model. Wilms tumor 1 (WT1) is an oncogenic zinc-finger transcription factor with low expression in the BM and notable overexpression in various hematological malignancies (AML and CLL), as well as in several solid tumors (such as glioblastoma, mesothelioma, and ovarian cancer).^385^

Moreover, LILRB4, a leukocytic immunoglobulin-like receptor belonging to the LILRB family,^386–389^ has shown promising results in preclinical studies.^389^ The LILRB family is expressed on AML cells and has been reported to be uniquely expressed on normal monocytic cells at the promonocyte stage of development.^390^ An interesting first-in-human study presented at the last ASH congress (San Diego, December 2024) demonstrated that LILRB4 STAR-T therapy is a promising approach in LILRB4 -positive R/R AML patients.^391^ Further data from the Phase II trial is eagerly awaited. Additionally, exciting findings were reported on a humanized IgG1 monoclonal antibody with high affinity and specificity for LILRB4, tested in combination with azacytidine for CMML. This combination appears to be well tolerated, with preliminary efficacy outcomes showing superiority over azacytidine alone, leading to rapid and sustained responses.^392^

Similarly, a potential new target for developing CAR-T cells in AML is CD84, an immunoreceptor belonging to the SLAM family (SLAMF5^393,394^). CD84 is overexpressed on AML cells while displaying comparatively low expression in CD34+ HSCs and absent in other tissues. Perez-Amill et al.^395^ developed a CD84-CAR-T therapy with promising in vitro and in vivo results.

Siglec-6 is expressed in primary AML blasts but is absent in normal HSCs.^396,397^ Building on this finding, Jetani et al.^397^ engineered a Siglec-6 CAR-T cell therapy utilizing a targeting domain derived from the mAb JML-1. Subsequent in vitro and in vivo studies demonstrated its remarkable efficacy against AML blasts while preserving regular HSCs activity. A similarly featured antigen is TIM-3 (T-cell immunoglobulin mucin-3), which is expressed in LSCs in almost all types of AML but not in HSCs.^398–400^ TIM3-CAR-T cells exhibited robust antileukemia activity in xenograft models, accompanied by the production of IFN-γ, granzyme B, and perforin.^224^

Furthermore, tumor cells are known to express cancer-specific surface protein conformations that are difficult to detect using standard technologies assessing gene or protein expression. However, these unique conformations can be identified and selectively targeted. In this context, recent studies have applied structural surfaceomics to AML, integrating cross-linking mass spectrometry with glycoprotein surface capture. Although still an emerging field, preliminary findings suggests that AML exhibits a distinct conformational signature involving various proteins, including integrin β2, which has been proposed as a potential target for CAR-T therapy.^401^

Another emerging approach that could circumvent the challenges in AML treatment is the combination of antibodies and cell-based therapies, known as STAb (Secreting T-cell-engaging Antibody). This strategy is based on the endogenous secretion of T cell-redirecting bispecific antibodies (bsAbs).^402^ STAb immunotherapies involve genetically modifying T cells with nucleic acids or viral vectors to encode bsAbs. This approach not only offers therapeutic potential similar to CAR-T cells but also enables the secreted bispecific antibodies to bind and activate bystander T cells. As a result, all circulating T cells are effectively “converted” into CAR-T-like cells, enhancing overall efficacy.^403,404^ The ability to recruit and activate bystander T cells amplifies the immune response, making STAbs a promising strategy for targeting cancer and other diseases by broadening immune activation beyond the directly engineered T cells.

This T cells engineered to produce bsAbs have demonstrated antitumor activity in preclinical models.^405^ For example, CD1a-, CD19-, and BCMA-STAb T cells have been tested in PDX models of cortical T-ALL, B-ALL and MM, respectively, showing higher efficacy than their respective second-generation CAR-based therapies.^406^ Similarly, dual-targeted STAb-T cells secreting BCMA TCE and CD19 TCE have been effectively tested in in vitro models of B-cell malignancies.^407^ This platform has also been studied in B-ALL, with promising results. An interesting example is a dual-target strategy based on T cells expressing an anti-CD22-CAR and also secreting an anti-CD19 T-cell engager antibody. This approach was compared with a previously validated anti-CD19/CD22 tandem CAR therapy, demonstrating that STAb-T cells exhibited an enhanced and faster in vitro cytotoxic activity.^408^ Given its ability to recruit bystander T cells, this innovative therapy is particularly promising for aggressive diseases prone to relapse with low effector T cells counts, such as AML. Indeed, some preliminary studies in AML have reported encouraging results.^409,410^

**Fig. 9:**
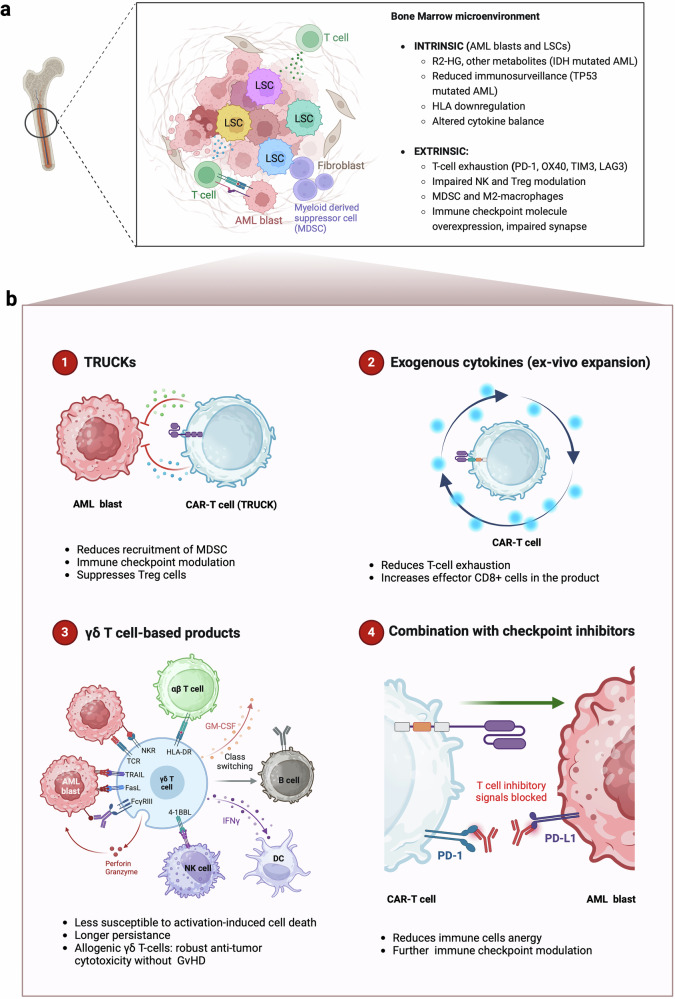
AML BM niche with intrinsic and extrinsic features (**a**) and future strategies to modulate tumor microenvironment (**b**). Among the promising strategies for modulating the bone marrow microenvironment, TRUCKs (1), the addition of exogenous cytokines to boost ex-vivo expansion (2), the use of gamma-delta T cell-based products (3), and the combination with checkpoint inhibitors (4) are especially noteworthy. Figure created with Biorender.com

There is mounting interest in gamma-delta (γδ) T cell-based products for adoptive immunotherapy.^411–414^ Specifically, Delta One T (DOT) cells (Vδ1 + γδ T cells) have been identified as a promising avenue for cancer immunotherapy due to their reduced susceptibility to activation-induced cell death and their capacity to persist as tumor-reactive lymphocytes over extended periods (Fig. 9).^415^ Preclinical studies have demonstrated the safety and efficacy of this approach,^416,417^ and it is undergoing testing in a Phase 1 clinical trial (NCT05015426) as a single infusion following alloHSCT.

### Modulating the tumor microenvironment

Given the T cell-inhibiting role of MDSCs,^418–421^ concomitant administration of CAR-T cells with small molecules capable of depleting the MDSC population may be synergistic.^422^ Several strategies that enhance CAR-T cell efficacy in the context of B-cell malignancies may also be relevant in AML. These include the upregulation of IL-15 expression or the IL-18 transgene (TRUCKs) and the addition of exogenous IL-7, IL-15, and/or IL-21 cytokines during the ex vivo expansion of CAR-T cells^423–426^ (Fig. 9).

Because of the low mutational burden of AML and the correspondingly low frequency of AML-reactive T cells,^427^ CAR-T cell combinations with checkpoint inhibitors (e.g., anti-PD-1 or anti-CTLA-4 antibodies) might be an interesting future strategy^428,429^ as it may also overcome T cell exhaustion and enhance CAR-T cell efficacy. This could be achieved either through exogenously administered inhibitors or genetic engineering, enabling the CAR-T cell to synthesize these antibodies.^430^ For example, the combination of immune checkpoint inhibitor PD-1/PD-L1 monoclonal antibodies and CAR-T cells has effectively prevented immune suppression and enhanced the anti-tumor activity of CAR-T cells.^431^ Similarly, novel strategies have been explored, such as the incorporation a Fc-mutant TIM3 receptor decoy to counteract inhibitory signals originating from the blasts or the TME in R/R B-ALL.^57^ In addition to TIM3, other immune checkpoint receptors, such as PD1, CLTA-4 and LAG3, can also be targeted as decoys to further disrupt these inhibitory signaling pathways. The flexibility to choose from a variety of inhibitory pathways to adapt this platform to different diseases offers a promising avenue for improving the overall efficacy and durability of CAR-based therapies.

The combined use of apoptosis-regulating drugs, such as BCL-2 inhibitors, can lower the threshold for CAR-T cells to trigger tumor cell killing through pathways like death receptors. This strategy helps overcome tumor cell resistance to therapy, thereby enhancing the overall effectiveness of the treatment.^432,433^ Moreover, combination with cytoreductive chemotherapy or epigenetic modulators such as HMA (e.g., azacitidine or decitabine) can reduce the tumor burden and modulate the immune microenvironment. There are several clinical trials ongoing testing the synergistic function of epigenetic drugs and immunotherapy.^434^ Indeed, it has been proven that CAR-T cells treated with methylation inhibitors such as low-dose decitabine during CAR T cell manufacturing present stronger antitumour effector function, proliferation, memory phenotype maintenance, cytokine release capacities and a lower exhaustion-associated gene expression under tumor cell stimulation in vivo and in vitro.^435^ These epigenetic agents before CAR-T cell infusion may enhance CAR-T efficacy by reducing immunosuppressive cells or increasing antigen expression on AML blasts.^77,435^ Moreover, some studies have used histone, DNA, and miRNA modifications to downregulate inhibitory molecules such as PD1, CTLA-3, TIM2 and LAG3 resulting in T cell-intrinsic upregulation.^436–438^ Additionally, the combination with immunomodulators like lenalidomide or TGF-β inhibitors can promote a more favorable microenvironment for CAR-T activity.^439,440^

Combining CAR-T cells with metabolic modulators also represents a promising strategy, as metabolic pathways are integral to T cells’ function, persistence, and efficacy. CAR-T cells, require robust metabolic activity to support their proliferation, survival, and effector functions. Within the TME, however, metabolic stressors such as hypoxia, nutrient deprivation, and the accumulation of immunosuppressive metabolites can lead to CAR-T cell exhaustion and dysfunction.^441^ In this context, there is some preclinical evidence of the combination of metabolic modulators with CAR-T cell therapy. For instance, increasing L-arginine levels (crucial for CAR-T cell proliferation and cytotoxicity) by modulating PRODH2 enzyme seems a preclinical exciting strategy.^442,443^ A study modified CAR-T cells to overexpress kynureninase, increasing their cytotoxicity in the TME.^444^ Further publications combined IDO1 inhibitors (a key enzyme in kynurenine synthesis) with CAR-T cells.

## Conclusions and perspectives

Modern CAR-T cell therapy represents the culmination of decades of immunology and genetic engineering research. Foundational discoveries, such as the identification of T cells and the demonstration of immune-mediated cancer eradication, as well as the early efforts led by Dr. Steven Rosenberg highlighting the potential of TILs, paved the way for advancements in adoptive cell therapy.

The first FDA-approved CAR-T therapies, Kymriah® and Yescarta®, revolutionized treatment for B-cell malignancies. Despite these successes, challenges remain, particularly in extending CAR-T therapy to solid tumors and certain hematological malignancies such as AML.

Regarding solid tumors, the tumor microenvironment, antigen heterogeneity, and limited CAR-T cell persistence present significant obstacles. This review describes that while no CAR-T therapy has received FDA approval for solid malignancies, certain strategies such as intracranial IL-13Ra-targeted CAR therapy (multifocal glioblastoma^124^), and ongoing clinical trials with Claudin18.2 (gastrointestinal tumors^126^) and GD2 (H3K27M-mutated glioma^125^; and neuroblastoma^127^) show promise.

Additionally, other T cell-based approaches, including ongoing trials targeting antigens like bispecific TCE and TCR-T therapies, are being explored in this specific solid tumor setting. For instance, the gp100 peptide-MHC/CD3 bispecific T-cell engager (TCE) tebentafusp, which was approved for uveal melanoma in 2022,^128^ is one strategy that holds great promise. In line with this, further approaches described in this review that are being currently tested show strong potential, such an autologous TCR T-cell therapy named afami-cel for synovial sarcoma,^129^ and other cellular therapies for HPV-associated cancers.^130–132^ While significant challenges remain, the evolving landscape of CAR-T cell therapy offers promising avenues for expanding its impact beyond hematologic malignancies.

Furthermore, CAR-T cell therapy holds great promise for the treatment of AML. However, several challenges must be addressed to fully realize its therapeutic potential. AML presents a highly heterogeneous landscape, driven by diverse genetic, cytogenetic, and epigenetic alterations that influence disease presentation, progression, and treatment response. The complexity of AML remains a challenge, particularly due to the presence of LSCs and clonal evolution mechanisms that contribute to therapy resistance and relapse.

Additionally, as exposed in this work, the AML bone marrow microenvironment further exacerbates these challenges by fostering immune evasion, metabolic suppression, and a highly immunosuppressive setting that impairs immune-based therapies. The BM microenvironment in AML is characterized by metabolic byproducts such as lactate, adenosine, and kynurenine, which inhibit T cell function and drive immune exhaustion. Furthermore, the presence of myeloid-derived suppressor cells (MDSCs), T regulatory cells (Tregs), and inhibitory checkpoints, such as PD-1, TIM-3, and LAG-3, contributes to T cell dysfunction. These immunosuppressive factors not only hinder the effectiveness of conventional therapies but also pose significant obstacles to CAR-T cell therapy, which has demonstrated limited success in AML compared to its applications in B-cell malignancies.

Moreover, a major limitation described in this review still to be faced in the development of CAR-T cell therapies for AML is the lack of an optimal target antigen. An ideal target should be highly expressed on AML blasts and LSCs while being absent in normal hematopoietic and extra-hematopoietic tissues to minimize on-target/off-tumor toxicity. Current CAR-T cell targets under investigation include CD123, CD33, CLL-1, NKG2D, CD7, CD38, CD44v6, CD70, and FLT3. Each of these antigens presents unique advantages and challenges, including concerns related to myeloablation, endothelial toxicity, and antigen escape mechanisms. Among these targets, CD123 and CD33 have been extensively studied, with various CAR-T cell constructs in preclinical and early clinical phases. However, toxicity concerns, particularly myelosuppression, remain a significant challenge. Emerging targets such as CLL-1 and NKG2D offer promising avenues due to their selective expression in AML while sparing normal HSCs. Additionally, strategies such as bispecific CARs, safety switches, and combinatorial approaches with immune checkpoint inhibitors or metabolic modulators are being explored to enhance CAR-T cell efficacy and safety.

Overall, while significant progress has been made in understanding AML heterogeneity and immune evasion, further research is required to optimize CAR-T cell strategies and overcome the inherent challenges of AML treatment. The integration of multi-targeting approaches, improved manufacturing techniques, and a deeper understanding of the BM microenvironment may ultimately enhance the therapeutic potential of CAR-T cells in AML.

Additionally, certain concerns regarding CAR-T cell therapy must be carefully evaluated in the context of AML. For instance, reports have highlighted potential long-term side effects, including the incidence of secondary myeloid neoplasms following CD19-CAR-T cell therapy.^445,446^ Studies estimate the incidence to range from 1% to 10%,^427–429^ which is comparable to the risk of therapy-related myeloid neoplasms observed after chemotherapy or autologous HSCT in patients with NHL. Furthermore, the FDA had raised concerns following the diagnosis of 22 cases of T cell malignancies within two years of CAR-T infusion.^447^ While these findings warrant attention, further studies and long-term follow-up are necessary to draw definitive conclusions regarding the risks associated with CAR-T cell therapy.^448^
